# Preparation of acute midbrain slices containing the superior colliculus and periaqueductal Gray for patch-clamp recordings

**DOI:** 10.1371/journal.pone.0271832

**Published:** 2022-08-11

**Authors:** Oriol Pavón Arocas, Tiago Branco

**Affiliations:** Sainsbury Wellcome Centre for Neural Circuits and Behaviour, University College London, London, United Kingdom; University of Texas Health Science Center, UNITED STATES

## Abstract

This protocol is a practical guide for preparing acute coronal slices from the midbrain of young adult mice for electrophysiology experiments. It describes two different sets of solutions with their respective incubation strategies and two alternative procedures for brain extraction: decapitation under terminal isoflurane anaesthesia and intracardial perfusion with artificial cerebrospinal fluid under terminal isoflurane anaesthesia. Slices can be prepared from wild-type mice as well as from mice that have been genetically modified or transfected with viral constructs to label subsets of cells. The preparation can be used to investigate the electrophysiological properties of midbrain neurons in combination with pharmacology, opto- and chemogenetic manipulations, and calcium imaging; which can be followed by morphological reconstruction, immunohistochemistry, or single-cell transcriptomics. The protocol also provides a detailed list of materials and reagents including the design for a low-cost and easy to assemble 3D printed slice recovery chamber, general advice for troubleshooting common issues leading to suboptimal slice quality, and some suggestions to ensure good maintenance of a patch-clamp rig.

## Introduction

The acute brain slice remains an essential preparation for studying the nervous system [1]. For decades, acute brain slices have been combined with the patch-clamp technique [2–4] for investigating the biophysical and electrophysiological properties of different cell types in a multitude of brain areas and animal species, including humans [5–10]. More recently, the refinement of viral and gene-editing approaches and the development of techniques such as optogenetics and single-cell transcriptomics have significantly expanded the range of questions that can be investigated with this preparation [11–27].

Despite the long tradition of patch-clamp experiments and the many adaptations of slicing protocol described in the literature [28–40], the success rate of the method and the quality of the tissue can vary depending on the brain region of interest and the age of the animal. Before starting a set of experiments in a brain area or animal age different from what is normally used in the home institution, it is important to test different combinations of solutions, brain extraction methods, and incubation strategies in order to find the approach that reliably provides the best tissue quality. Here, we describe a step-by-step guide for obtaining acute coronal slices from the midbrain of young adult mice (4–12 weeks old). This includes instructions for preparing solutions and extracting the brain, a detailed list of materials and reagents, and troubleshooting advice.

## Materials and methods

The protocol described in this peer-reviewed article is published on protocols.io, https://dx.doi.org/10.17504/protocols.io.8epv51pz5l1b/v6 and is included for printing as S1 File with this article.

## Expected results

The protocol described here has been routinely used to obtain patch-clamp recordings from midbrain neurons in acute slices containing the superior colliculus (SC) and periaqueductal gray (PAG) for investigating their biophysical properties and their role within the neural circuits controlling instinctive behaviours [18, 22, 25].

**Fig 1:**
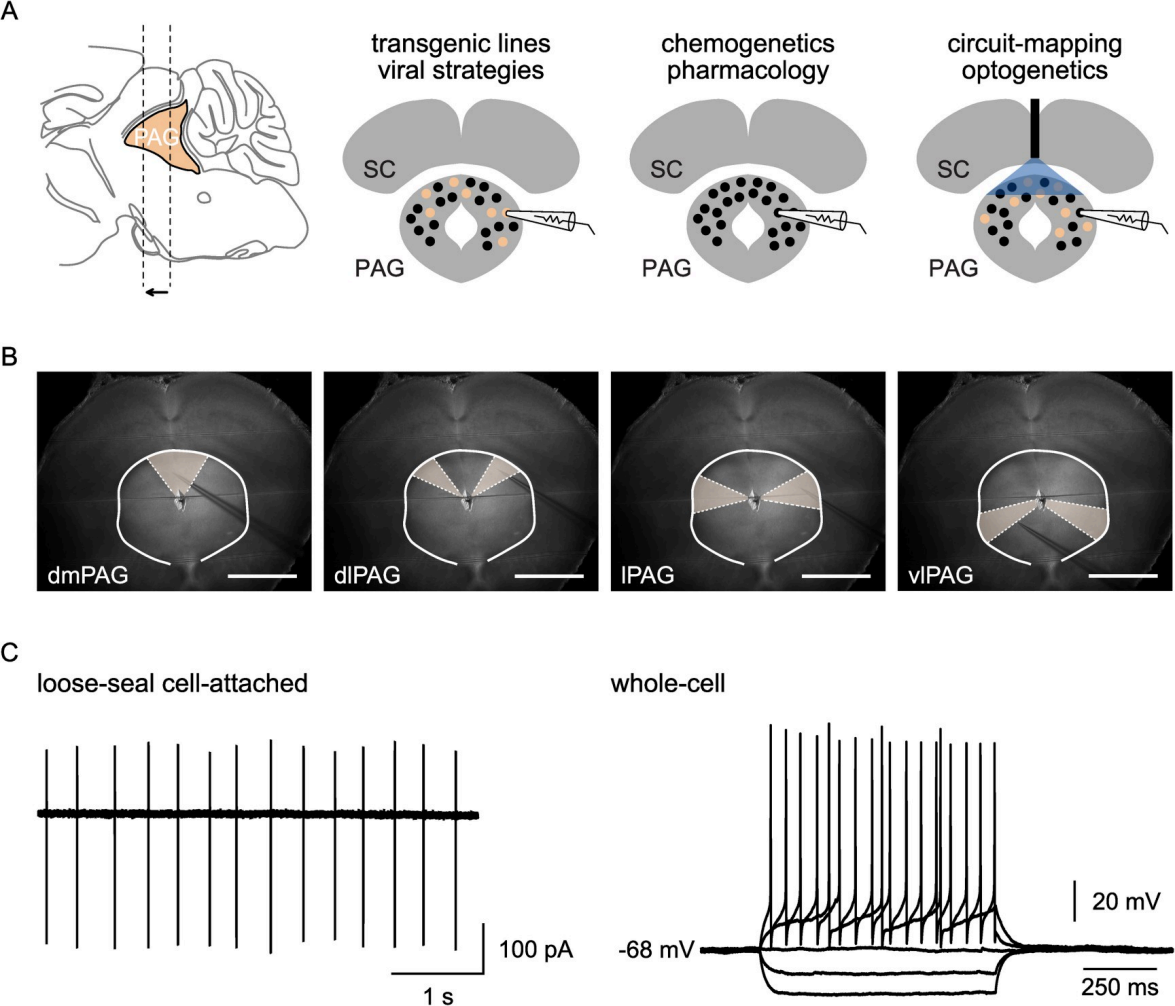
Targeted patch-clamp recordings in acute midbrain slices. A. Schematics illustrating the use of acute midbrain slices in combination with other techniques to target neurons in a midbrain area of interest (PAG, in yellow). B. Example images from experiments targeting midbrain neurons in different anatomical subdivisions of the PAG in the same slice. Scale bars are 1 mm. Abbreviations indicate the different PAG subdivisions: dorsomedial (dmPAG), dorsolateral (dlPAG), lateral (lPAG), and ventrolateral (vlPAG). C. (Left) Spontaneous electrical activity of a PAG neuron during a loose-seal cell-attached recording. (Right) Voltage response to step current injections of a PAG neuron during a whole-cell recording.

This method can be combined with the use of transgenic lines or viral strategies to target genetically labelled subsets of neurons in the midbrain area of interest (Fig 1A) or any of its anatomical subdivisions (Fig 1B). Furthermore, patch-clamp recordings in the cell-attached or whole-cell configuration (Fig 1C) can be obtained while pharmacological, optogenetic, or chemogenetic manipulations are applied to the slice (Fig 1A).

## Supporting information

S1 File Preparation of acute midbrain slices containing the superior colliculus and periaqueductal Gray for patch-clamp recordings. Step-by-step protocol, also available on protocols.io.
(PDF)
