# Supplementary material for: Preparation of acute midbrain slices containing the superior colliculus and periaqueductal Gray for patch-clamp recordings
Source: PLoS One. 2022 Aug 11;17(8):e0271832. doi: 10.1371/journal.pone.0271832 (PMC9371254; doi:10.1371/journal.pone.0271832)
Supplement: S1 File — Step-by-step protocol, also available on protocols.io. (PDF) [file pone.0271832.s001.pdf]

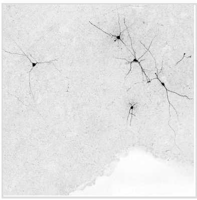

Version 6

Jun 30, 2022

Preparation of acute midbrain slices containing the superior colliculus and periaqueductal gray for patch-clamp recordings V.6

Oriol Pavon Arocas[1], Tiago Branco[1]

[1]Sainsbury Wellcome Centre for Neural Circuits and Behaviour, University College London, Howland Str eet, London, W1T 4JG, UK

1 Works for me Share

dx.doi.org/10.17504/protocols.io.8epv51pz5l1b/v6

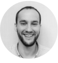

Oriol Pavon
Sainsbury Wellcome Centre for Neural Circuits and Behaviour

DISCLAIMER

This work was funded by a Wellcome Senior Research Fellowship (214352/Z/18/Z) and a Sainsbury Wellcome Centre Core Grant from the Gatsby Charitable Foundation and Wellcome (090843/F/09/Z) (T.B.), and by a UCL Wellcome 4-year PhD Programme in Neuroscience Fellowship (203729/Z/16/Z) (O.P.A.).

We would like to thank Nicolas Wanaverbecq, Annalisa Scimemi, Vanessa Stempel, Yaara Lefler, Yu Lin Tan, Simon Weiler, Sara Mederos, and Hedi Young for their help and advice prior to the development of this protocol. We would also like to thank Robb Barrett from the Fabrications Lab at SWC for designing the 3D printed piece for the recovery chamber.

ABSTRACT

This protocol is a practical guide for preparing acute coronal slices from the midbrain of young adult mice for electrophysiology experiments. It describes two different sets of solutions with their respective incubation strategies and two alternative procedures for brain extraction: decapitation under terminal isoflurane anaesthesia and intracardial perfusion with artificial cerebrospinal fluid under terminal isoflurane anaesthesia. Slices can be prepared from wild-type mice as well as from mice that have been genetically modified or transfected with viral constructs to label subsets of cells. The preparation can be used to investigate the electrophysiological properties of midbrain neurons in combination with pharmacology, opto- and chemogenetic manipulations, and calcium imaging; which can be followed by morphological reconstruction, immunohistochemistry, or single-cell transcriptomics. The protocol also provides a detailed list of materials and reagents including the design for a low-cost and easy to assemble 3D printed slice recovery chamber, general advice for troubleshooting common issues leading to suboptimal slice quality, and some suggestions to ensure good maintenance of a patch-clamp rig.

Citation: Oriol Pavon Arocas, Tiago Branco Preparation of acute midbrain slices containing the superior colliculus and periaqueductal gray for patch-clamp recordings https://dx.doi.org/10.17504/protocols.io.8epv51pz5l1b/v6

This is an open access protocol distributed under the terms of the **Creative Commons Attribution License** (https://creativecommons.org/licenses/by/4.0/), which permits unrestricted use, distribution, and reproduction in any medium,

DOI

dx.doi.org/10.17504/protocols.io.8epv51pz5l1b/v6

PROTOCOL CITATION

Oriol Pavon Arocas, Tiago Branco 2022. Preparation of acute midbrain slices containing the superior colliculus and periaqueductal gray for patch-clamp recordings. **protocols.io** https://dx.doi.org/10.17504/protocols.io.8epv51pz5l1b/v6
Version created by Oriol Pavon

KEYWORDS

Mouse, Slice electrophysiology, Patch-Clamp, Neuroscience, Acute Brain Slices, Midbrain, Superior Colliculus, Periaqueductal Gray

LICENSE

This is an open access protocol distributed under the terms of the Creative Commons Attribution License, which permits unrestricted use, distribution, and reproduction in any medium, provided the original author and source are credited

IMAGE ATTRIBUTION

Oriol Pavon Arocas

CREATED

Jun 30, 2022

LAST MODIFIED

Jun 30, 2022

PROTOCOL INTEGER ID

65674

GUIDELINES

A typical experiment could be scheduled as follows: preparation of acute slices in the morning, incubation steps during lunch break, and data acquisition using patch-clamp recordings for as long as the tissue remains viable.

MATERIALS TEXT

Tools for brain extraction and slicing:

- Metal box to store tools: Moria Instrument Case (Cat. No. 20311-21, InterFocus/Fine Science Tools, £75).
- Scissors for decapitation: Student Surgical Scissors Straight Sharp-Blunt, 14.5 cm (Cat. No. 91401-14, InterFocus/Fine Science Tools, £21.5).
- Fine scissors for skin: Extra Fine Bonn Scissors Straight, 8.5 cm (Cat. No. 14084-08, InterFocus/Fine Science Tools, £67).

Citation: Oriol Pavon Arocas, Tiago Branco Preparation of acute midbrain slices containing the superior colliculus and periaqueductal gray for patch-clamp recordings https://dx.doi.org/10.17504/protocols.io.8epv51pz5l1b/v6

This is an open access protocol distributed under the terms of the **Creative Commons Attribution License** (https://creativecommons.org/licenses/by/4.0/), which permits unrestricted use, distribution, and reproduction in any medium,

- Small serrated scissors for skull: Vannas Tübingen Spring Scissors Straight Serrated, 8.5 cm (Cat. No. 15070-08, InterFocus/Fine Science Tools, £242).
- Fine tweezers to detach meninges from brain: Dumont #5 Fine Forceps Biology Inox, (Cat. No. 11254-20, InterFocus/Fine Science Tools, £42).
- Forceps to separate skull: Micro-Adson Forceps Serrated (Cat. No. 11018-12, InterFocus/Fine Science Tools, £34).
- Blade to cut frontal part of the brain and cerebellum: Scalpel Handle #4 (Cat. No. 10004-13, InterFocus/Fine Science Tools, £12) and sterile blades (e.g. 24, Swann Morton).
- Spatula to remove brain from skull: Probe and Spatula, 14 cm (Cat. No. 10090-13, InterFocus/Fine Science Tools, £83.5).
- Spoon to immerse brain in ACSF slush: Moria MC17 Perforated Spoon, 20 mm tip diameter (Cat. No. 10370-17, InterFocus/Fine Science Tools, £136).
- Syringe and needle to separate and mark cut slices from brain: 1 mL syringe with a stainless steel needle (e.g. brown - BD Microlance 3, 26G, 0.45x13 mm, Cat. No. 303800). Can be carefully washed and reused.
- Brush to move and reposition the slices: Major Brushes size 2.
- Superglue (Universal, Loctite) to glue the brain on the magnetized block of the vibratome.
- Double edge blades for vibratome (Cat. No. 74-0002, Personna).
- Cotton buds and Acetone to clean the double edge blades before slicing.
- Filter paper, 70 mm diameter (Cat. No. 11556873, Fisher).
- Glass Pasteur pipette to make a slice collector to pick and transport brain slices (Cat. No. D810, Poulten & Graf).
- Ice Pan, 2.5 L (Cat. No. 12318687, Bel-Art).
- Disposal bag (Cat. No. 86.1197, Sarstedt).
- Plastic tray, silicon mat, 20 mL syringe, and sterile stainless steel needles for fixing the mouse during intracardial perfusion.

Glassware and other tools for preparing solutions and brain extraction:

- Volumetric flasks for solutions: class A NS 24/29 1000 mL PE (Cat. No. 612-3819, VWR) and class A NS 29/32 2000 mL PE (Cat. No. 612-3820, VWR).
- Magnetic stir bars (Cowie Technology PTFE Cylindrical Stir Bar Box Set, £33).
- Glass beaker, 400 mL, for slicing solution (Borosilicate glass with spout, Cat. No. 213-0046, VWR).
- Glass beaker, 150 mL, one for each recovery chamber (Borosilicate glass with spout, Cat. No. 213-1123, VWR).
- Glass beaker, 50 mL, to collect the head after decapitation (Borosilicate glass with spout, Cat. No. 213-1121, VWR).
- Crystallizing dish without spout, 150 mL, Duran Borosilicate Glass, 80 mm diameter, 45 mm height (Cat. No. 11786582, Fisher Scientific UK).
- Petri dish, Duran Glass, 100 mm diameter, 20 mm height (Cat. No. 10796771, Fisher Scientific UK).
- Glass bottles, 250 mL, DIN thread with cap (Cat. No. 215-1515, VWR).
- Falcon tube, 50 mL.
- Microfilter candles to diffuse carbogen into solutions: 6 mm diameter, porosity P3, narrow glass tube (Cat. No. ROBU18103, Robuglas).
- Ice-Cream Maker to prepare slush (Magimix).
- Blender.
- Spatulas and plastic containers to weigh salts.

Citation: Oriol Pavon Arocas, Tiago Branco Preparation of acute midbrain slices containing the superior colliculus and periaqueductal gray for patch-clamp recordings https://dx.doi.org/10.17504/protocols.io.8epv51pz5l1b/v6

This is an open access protocol distributed under the terms of the **Creative Commons Attribution License** (https://creativecommons.org/licenses/by/4.0/), which permits unrestricted use, distribution, and reproduction in any medium,

- Funnel.
- Tape to label instruments and glassware.
- Clamp hoses for up to 10 mm diameter tubing (Cat. No. 229-0609, Buerkle).

Optional:

- Repair Kit for Dumont Forceps (Cat. No. 29000-00, InterFocus/Fine Science Tools, £74.5).
- Instrument Tip Protectors (Cat. No. 29060-00, InterFocus/Fine Science Tools, £15).

Salts for preparing solutions:

- Sodium Chloride (NaCl, Cat. No. S7653-1KG, Sigma).
- N-Methyl-D-Glucamine (NMDG, Cat. No. 66930-500G, Sigma).
- Potassium Chloride (KCl, Cat. No. P9333-500G, Sigma).
- Sodium Phosphate Monobasic (NaH_2PO_4, Cat. No. S8282-500G, Sigma).
- Sodium Bicarbonate ($NaHCO_3$, Cat. No. S6297-250G, Sigma).
- 4-(2-hydroxyethyl)-1-piperazineethanesulfonic acid (HEPES, Cat. No. H3375-500G, Sigma).
- D-Glucose (Cat. No. G7528-250G, Sigma).
- Sucrose (Cat. No. S0389-500G, Sigma).
- Sodium L-Ascorbate (Cat. No. 11140-250G, Sigma).
- Thiourea (Cat. No. T7875-100G, Sigma).
- Sodium Pyruvate (Cat. No. P2256-100G, Sigma).
- Myo-Inositol (Cat. No. I5125-500G, Sigma).
- N-Acetyl-L-Cysteine (Cat. No. A9165-100G, Sigma).
- L-Ascorbic Acid (Cat. No. A92902-100G, Sigma).
- Taurine (Cat. No. 93019-100MG, Sigma).
- Calcium Chloride ($CaCl_2$, 1 M solution, Cat. No. 21114-1L, Honeywell Fluka).
- Magnesium Chloride ($MgCl_2$, 1 M solution, Cat. No. 63020-1L, Honeywell Fluka).
- Magnesium Sulfate Heptahydrate ($MgSO_4.7H_2O$, Cat. No. 63138-250G, Sigma).
- Sodium Hydroxide (NaOH, 5 M solution, Cat. No. S8263-150ML, Sigma).
- Hydrochloric Acid (HCl, 5 M solution, Cat. No. 10615882, J/4310/17, Fisher).

Instruments for preparing solutions and slicing:

- Ultrapure ddH_2O (or purification system to obtain it) to prepare solutions and clean glassware. In case of doubt, purchase Molecular Biology Grade Water that is autoclaved sterile, reverse osmosis purified to 18 MOhms, bacterial filtered 0.2 microns, DNAse/RNAse/Protease free (Cat. No. 20-9103-00, Severn Biotech).
- Carbogen source.
- Precision scale.
- Micropipettes (1000 µL, 250 µL, 100 µL, 10 µL).
- Electronic pipette Easypet 3 (Cat. No. 4430000018, Eppendorf, £324).
- Mini vortex mixer (VWR).
- pH meter (Mettler Toledo).
- Osmometer and Sample discs (Cat. No. SS-033, ELITech Group Biomedical Systems).
- Osmolality Standard Vials to calibrate osmometer: Optimole 100 mmol/kg (Cat. No. NC9041262, Fisher), 290 mmol/kg (Cat. No. NC9306976, Fisher), 1000 mmol/kg (Cat. No. NC9306975,

Citation: Oriol Pavon Arocas, Tiago Branco Preparation of acute midbrain slices containing the superior colliculus and periaqueductal gray for patch-clamp recordings https://dx.doi.org/10.17504/protocols.io.8epv51pz5l1b/v6

This is an open access protocol distributed under the terms of the **Creative Commons Attribution License** (https://creativecommons.org/licenses/by/4.0/), which permits unrestricted use, distribution, and reproduction in any medium,

Fisher), from ELITech Group Biomedical Systems.
- Fridge and Freezer to store solutions and some of the components needed to prepare them.
- Heating blocks (QBD4, Grant Instruments) for bringing solutions to a specific temperature.
- Ventilation hood and anaesthetic chamber with isoflurane.
- Vibratome (Leica VT1200S or Campden Ci7000smz-2).
- Plastic bottles with ddH2O or 70% ethanol solution for cleaning.
- Rack for drying glassware.
- Oven to bake and sterilize glassware (Binder Oven E28, Wolf Labs, £544).
- NIGHTSEA Dual Fluorescent Protein Flashlight - Cyan and Green LEDs, to visualize expression of viral injections in acute slices (Cat. No. DFP-CG, CN Technical Services Ltd, £795).

Recovery chambers for incubating acute slices:

- Plastipak luer slip syringe (2 mL, sterile, Cat. No. BD574 or BDS0003, Appleton Woods).
- Sterile gauze swabs (10x10 cm, 8 ply, Cat. No. MSPPEJE136 or IBSA25071, VWR).
- Glass beaker, 150 mL, 60 mm external diameter (Borosilicate glass with spout, Cat. No. 213-1123, VWR).
- Double threaded plastic cap (Samco™ Narrow Mouth Bio-Tite™, 90 mL, 48 mm Specimen Containers, Sterile, Cat. No. 020039, Thermo Fisher).
- If plastic caps of the correct size can't be sourced, Robb Barrett from the Fabrications Lab at the Sainsbury Wellcome Centre (UCL) designed a 3D printed alternative - the relevant .stl and .step files to 3D print the pieces are available on GitHub (https://github.com/opavon/AcuteBrainSlices_RecoveryChamber).

Other instruments useful for patch-clamp recordings (not covered here):

- Borosilicate standard wall with filament capillary glass, 1.5 mm outer diameter, 0.86 mm inner diameter, 100 mm length (Cat. No. 300057 - GC150F-10, Pack of 225 capillary glass, Harvard Apparatus via MultiChannel Systems, €46 each pack).
- PC-100 Micropipette Puller (Narishige via Digitimer, £2,289) for pulling patch pipettes.
- Pipette Holder (new BNC-type, 1.5 mm outer diameter, Cat. No. 895229 HEKA), Electrode Connector Pin (Cat. No. 895226, HEKA), back end O-Ring (pack of 10, Cat. No. 895232, HEKA), Holder replacement cap set containing 1x Screw Cap, 1x Distance Sleeve, 2x O-rings (for 1.5 mm outer diameter, Cat. No. 895098, HEKA), O-rings for Cap (pack of 10, Cat. No. 895017, HEKA), Ground Connector Pin (pack of 10, Cat. No. 895104, HEKA).
- Silver wire, 0.35 mm diameter, 5 m length (Cat. No. AG005145, GoodFellow, £95).
- Ag-AgCl pellet and wire electrodes: Model E206 pellet electrode 2.0 mm diameter, Electrode: 2.0 x 2.5 mm (diameter x length) - Wire: 0.25 x 70 mm (diameter x length) (Cat. No. 641310, Warner Instruments via MultiChannel Systems, €20 each).
- Micropipette 2-20 µL (Cat. No. 17014412, Mettler Toledo, Rainin) and Microloader Tips (Cat. No. 5242956003, Eppendorf) for backfilling patch pipettes with intracellular solution prior to recording.
- Platinum wire to make the harps to hold the slices in the recording chamber, 0.1 m length, 1 mm diameter, High Purity: 99.99+%, Temper: As drawn (Cat. No. 038-889-51, PT005155 Platinum Wire, Goodfellow, £299).
- SliceScope Pro 1000 or alternative (Scientifica).
- pE-100 LED, 490 nm and 565 nm, to visualize EYFP and tdTomato in the slices (CoolLED via Scientifica, £850).

Citation: Oriol Pavon Arocas, Tiago Branco Preparation of acute midbrain slices containing the superior colliculus and periaqueductal gray for patch-clamp recordings https://dx.doi.org/10.17504/protocols.io.8epv51pz5l1b/v6

This is an open access protocol distributed under the terms of the **Creative Commons Attribution License** (https://creativecommons.org/licenses/by/4.0/), which permits unrestricted use, distribution, and reproduction in any medium,

- Motorised micromanipulator (PatchStar, Scientifica).
- Patch-clamp headstage and amplifier (EPC 800, HEKA).
- PCIe-6353 board (National Instruments).
- CMOS Camera, USB3.0 Industrial 1.3MP B/W Camera (E2V) EV76C560AB, global shutter (Cat. No. MQ013MGE2, Lambda Photometrics Ltd, £355).
- Micrometers to calibrate the pixel size of the images obtained with the mounted camera: 20 mm microscope scale with crossline (Cat. No. R1L1S5P, ThorLabs), 10 mm Stage Micrometer with 50 µm Divisions (Cat. No. R1L3S1P, ThorLabs), 1 mm Stage Micrometer with 10 µm Divisions (Cat. No. R1L3S2P, ThorLabs).
- Copper Mesh (Cat. No. PSY406, Thorlabs) and Neodymium Magnetic Rings with 20 mm hole (Cat. No. 792-4565, Eclipse via RS components) to cover the faraday cage of the rig. Needs to be grounded.
- Persistaltic perfusion system PPS2 (MultiChannel Systems).
- Tubing for the Scientifica Heater (the one that coils on top of the heating element): Polyethylene tubing, PE-10, 100 feet (Cat. No. 640751, Multichannel Systems).
- Manometer to monitor the air pressure applied to the patch pipette (AP-34, Keyence).
- DS3 Isolated Current Stimulator (Digitimer, £735.2) and Electrode Holder (Digitimer, Model H-13).

SAFETY WARNINGS

This protocol involves animal work and potential exposure to allergens. Wear appropriate personal protective equipment (PPE) and follow all the protocols in place at your home institution.

DISCLAIMER:

This work was funded by a Wellcome Senior Research Fellowship (214352/Z/18/Z) and a Sainsbury Wellcome Centre Core Grant from the Gatsby Charitable Foundation and Wellcome (090843/F/09/Z) (T.B.), and by a UCL Wellcome 4-year PhD Programme in Neuroscience Fellowship (203729/Z/16/Z) (O.P.A.).

We would like to thank Nicolas Wanaverbecq, Annalisa Scimemi, Vanessa Stempel, Yaara Lefler, Yu Lin Tan, Simon Weiler, Sara Mederos, and Hedi Young for their help and advice prior to the development of this protocol. We would also like to thank Robb Barrett from the Fabrications Lab at SWC for designing the 3D printed piece for the recovery chamber.

BEFORE STARTING

Ensure any animal work is carried out following the legislation and protocols in place at your home institution.

General considerations

1

This protocol provides a step-by-step guide for obtaining acute coronal slices from the midbrain of young adult mice (4-12 weeks old). The following sections include instructions for preparing two different sets of solutions with their respective incubation strategies and two alternative procedures for brain extraction: decapitation under terminal isoflurane

Citation: Oriol Pavon Arocas, Tiago Branco Preparation of acute midbrain slices containing the superior colliculus and periaqueductal gray for patch-clamp recordings https://dx.doi.org/10.17504/protocols.io.8epv51pz5l1b/v6

This is an open access protocol distributed under the terms of the **Creative Commons Attribution License** (https://creativecommons.org/licenses/by/4.0/), which permits unrestricted use, distribution, and reproduction in any medium,

anaesthesia (Path A) and intracardial perfusion with artificial cerebrospinal fluid (ACSF) under terminal isoflurane anaesthesia (Path B).

- Path A is the simpler of the two approaches: the solutions are easier to prepare and the brain extraction is preceded by terminal anaesthesia and decapitation. This makes Path A less technically challenging than Path B.

- Path B is more elaborated: it requires three solutions instead of two, there is an additional incubation step, and the decapitation and brain extraction are preceded by intracardial perfusion. Intracardially perfusing the animal with ice-cold slicing solution before extracting the brain is the best way to remove the blood out of the tissue by replacing it with the solution of your choice (e.g. low sodium and calcium and high magnesium and NMDG) to rapidly slow metabolic activity and reduce excitotoxicity.

Given its easier implementation, our recommendation is to initially follow Path A and assess the quality of the resulting slices. In our hands, this method provides good quality slices especially when used in younger animals (up to 7-8 weeks old). If the quality of the slices is found to be suboptimal, or if the experimental design requires using animals that have undergone stereotaxic surgery or are >8 weeks old, our recommendation is to implement Path B and read Section 8 (Troubleshooting slice quality problems) for further suggestions. We have found that both the solutions (NMDG-HEPES slicing and HEPES holding) and the intracardial perfusion method used in Path B significantly improve the quality of the slices and the amount of healthy cells found in the tissue.

1.1 Abbreviations

ACSF, artificial cerebrospinal fluid
AgCl, silver chloride
CaCl2, calcium chloride
cm, centimetre
CO2, carbon dioxide
ddH2O , double distilled water
g, gram
HCl, hydrochloric acid
HEPES, 4-(2-hydroxyethyl)-1-piperazineethanesulfonic acid
KCl, potassium chloride
kg, kilogram
L, litre
M, molar
m, metre
MgCl2, magnesium chloride
MgSO4, magnesium sulfate
min, minute
mL, millilitre
mL/min, millilitre per minute
mm, millimetre

Citation: Oriol Pavon Arocas, Tiago Branco Preparation of acute midbrain slices containing the superior colliculus and periaqueductal gray for patch-clamp recordings https://dx.doi.org/10.17504/protocols.io.8epv51pz5l1b/v6

This is an open access protocol distributed under the terms of the **Creative Commons Attribution License** (https://creativecommons.org/licenses/by/4.0/), which permits unrestricted use, distribution, and reproduction in any medium,

mM, millimolar
mOsm/kg, milliosmoles per kilogram
MW, molecular weight
NaCl, sodium chloride
NaHCO3, sodium bicarbonate
NaH2PO4, sodium monobasic phosphate
NaOH, sodium hydroxide
nm, nanometre
NMDG, N-Methyl-D-Glucamine
NMDG-Cl, N-Methyl-D-Glucamine chloride
O2, oxygen
PAG, periaqueductal gray
PFA, paraformaldehyde
PPE, personal protective equipment
°C, degrees Celsius
µL, microlitre
µm, micrometre

Preparation of stock solutions 2h

2

This section describes two different sets of solutions for two different methods for slicing.

Path A uses a sucrose-based slicing solution in which a proportion of NaCl has been replaced by sucrose. This reduces the sodium driving force, ensuring there is less entry of sodium into the cells and reducing excitotoxicity. Sodium phosphate and sodium bicarbonate are buffers that help maintain the pH within a physiological range. The low calcium / high magnesium ratio in the slicing solution reduces neurotransmitter release and activation of NMDA receptors, and thus maintains a low overall excitability of the brain tissue.

The slicing solution in Path B has been optimised for adult brain tissue (Ting, et al., 2018). Using NMDG-Cl to completely replace NaCl helps eliminates sodium entry into the cells, which together with a low temperature and intracardial perfusion slows down metabolism and reduces excitotoxicity even more. The addition of 20 mM HEPES effectively provides stronger pH buffering in the physiological range and prevents excessive edema. Myo-inositol and sodium pyruvate provide additional entry points into cellular metabolism, whereas ascorbic acid, thiourea, and N-acetyl-L-cysteine (a cell-permeable glutathione precursor) help reduce oxidative stress.

In both cases, it is important to use purified water that is free of contaminants such as trace metals. Impurities like these are often found in distilled water sources and cause damage to the slices and can convert anti-oxidants such as ascorbate into pro-oxidants.

Besides the composition of the slicing solution, the other main difference between paths A and B is that path B uses a holding solution where slices are kept until used for recording. The holding solution effectively acts as a bridge between the very protective properties of the NMDG-based slicing solution and the physiological concentrations of the recording

Citation: Oriol Pavon Arocas, Tiago Branco Preparation of acute midbrain slices containing the superior colliculus and periaqueductal gray for patch-clamp recordings https://dx.doi.org/10.17504/protocols.io.8epv51pz5l1b/v6

This is an open access protocol distributed under the terms of the **Creative Commons Attribution License** (https://creativecommons.org/licenses/by/4.0/), which permits unrestricted use, distribution, and reproduction in any medium,

solution. Together, all these improvements make Path B the most protective of both approaches.

2.1 [PATH A - Decapitation under terminal isoflurane anaesthesia]

Path A follows the first of two alternative slicing methods. Briefly, it consists on decapitation under terminal isoflurane anaesthesia, brain extraction and slicing in ice-cold slicing ACSF, incubation in slicing ACSF for 30 min at 35°C, followed by incubation in recording ACSF for 30 min at room temperature, and finally transferral of a slice to the recording chamber perfused with recording ACSF at 32-34°C or at room temperature.

In general, preparing 2 L of each of the following solutions should be enough for four days of experiments. Once prepared, store the solutions in the fridge at 4°C and try not to use them if older than a week. Make sure to use ultrapure ddH_2O and to sterilise all glassware by baking for 2 hours at 200°C the previous day.

Slicing ACSF (sucrose)

For each experiment you will need ~500 mL of solution: ~150 mL for the first recovery chamber and ~350 mL to make the slush used during the slicing steps (see Section 3 for more details).

- Slicing ACSF (in mM): 87 NaCl, 2.5 KCl, 26 $NaHCO_3$, 1.25 NaH_2PO_4, 10 D-glucose, 50 sucrose, 0.5 $CaCl_2$, 3 $MgCl_2$ (equilibrated with 95% O_2 and 5% CO_2), with an osmolality of ~280 mOsm/kg.
- Optionally, you can add (in mM): 2 ascorbic acid, 2 sodium pyruvate, and 3 myo-inositol. Ascorbic acid is a free radical scavenger, while sodium pyruvate and myo-inositol are additional energy sources and entry points into cellular metabolism for the slices. If added, osmolality will increase by ~10 mOsm/kg.
- Equilibrate with 95% O_2 and 5% CO_2 and adjust pH to 7.3-7.4 before adding the $CaCl_2$ and $MgCl_2$.
- If you need to reduce/increase osmolality, reduce/increase the amount of sucrose to avoid modifying the other ion concentrations.

Citation: Oriol Pavon Arocas, Tiago Branco Preparation of acute midbrain slices containing the superior colliculus and periaqueductal gray for patch-clamp recordings https://dx.doi.org/10.17504/protocols.io.8epv51pz5l1b/v6

This is an open access protocol distributed under the terms of the **Creative Commons Attribution License** (https://creativecommons.org/licenses/by/4.0/), which permits unrestricted use, distribution, and reproduction in any medium,

Path A - Slicing ACSF (sucrose)							
for brain extraction and tissue slicing at 0-4°C							
for incubating slices at 35°C for 30 min							
after incubation, let slices cool to room temperature or transfer to recording ACSF at room temperature							
	**mM**	**g/L**	**g/2L**		**MW**	**Cat. No.**	**Company**
NaCl	87	5.084	**10.169**		58.44	S7653-1kg	Sigma
KCl	2.5	1 mL	**2 mL**	**(2.5 M stock)**	74.55	P9333-500g	Sigma
$NaHCO_3$	26	2.184	**4.369**		84.01	S6297-250g	Sigma
NaH_2PO_4	1.25	1 mL	**2 mL**	**(1.25 M stock)**	119.98	S8282-500g	Sigma
D-Glucose	10	1.802	**3.603**		180.16	G7528-250g	Sigma
Sucrose	50	17.115	**34.230**		342.3	S0389-500g	Sigma
$CaCl_2$	0.5	0.5 mL	**1 mL**	**(1 M stock)**	110.98	21114-1L	Honeywell Fluke
$MgCl_2$	3	3 mL	**6 mL**	**(1 M stock)**	95.21	63020-1L	Honeywell Fluke
Ascorbic acid	*2*	*0.3522*	***0.7045***		*176.12*	*A92902-100G*	*Sigma*
Sodium pyruvate	*2*	*0.2201*	***0.4402***		*110.04*	*P2256-100G*	*Sigma*
Myo-inositol	*3*	*0.5405*	***1.0810***		*180.16*	*I5125-500G*	*Sigma*

Always start with ultrapure ddH_2O amounting to ~80% of the target volume.
Add all ingredients except for the divalents (Ca^{2+} and Mg^{2+}).
Adjust pH to 7.3-7.4 with NaOH or HCl if necessary. Once the pH is in range, add Ca^{2+} and Mg^{2+}.
Top off the volume with ultrapure ddH_2O.

Check osmolality: ~290 mOsm/kg (if adding the components in italics, otherwise ~280 mOsm/kg).
Ascorbic acid is a free radical scavenger.
Sodium pyruvate and Myo-inositol are additional energy sources that help cellular metabolism.

Prior to use, equilibrate the solution with 95% O_2, 5% CO_2 for at least 30 min.
Adjust pH again right before use (it gets acidic when cold).

Figure S1. Recipe for Slicing ACSF (sucrose).

Recording ACSF

For each experiment you will need ~500 mL of solution: ~150 mL for the second recovery chamber and ~250 mL for superfusing the slices during recording. These values are given as an approximation under the assumption that you recirculate the solution and do not need to wash in specific compounds. If you do not recirculate the solution or need to test the effect of washing in a specific compound, you will need more solution for each experiment.

- Recording ACSF (in mM): 125 NaCl, 2.5 KCl, 26 NaHCO3, 1 NaH2PO4, 10 D-glucose, 2 CaCl2, and 1 MgCl2 (equilibrated with 95% O2 and 5% CO2), with an osmolality of ~295 mOsm/kg.
- Optionally, you can add (in mM): 2 ascorbic acid, 2 sodium pyruvate, and 3 myo-inositol. Ascorbic acid is a free radical scavenger, while sodium pyruvate and myo-inositol are additional energy sources and entry points into cellular metabolism for the slices. If added, osmolality will increase by ~10 mOsm/kg.
- Equilibrate with 95% O2 and 5% CO2 and adjust pH to 7.3-7.4 before adding the CaCl2 and MgCl2.

Citation: Oriol Pavon Arocas, Tiago Branco Preparation of acute midbrain slices containing the superior colliculus and periaqueductal gray for patch-clamp recordings https://dx.doi.org/10.17504/protocols.io.8epv51pz5l1b/v6

This is an open access protocol distributed under the terms of the **Creative Commons Attribution License** (https://creativecommons.org/licenses/by/4.0/), which permits unrestricted use, distribution, and reproduction in any medium,

Path A - Recording ACSF

for incubating slices at room temperature for 30 min after sucrose incubation and before recording
for recording at 32-34°C or room temperature

	mM	g/L	g/2L		MW	Cat. No.	Company
NaCl	125	7.3050	**14.6100**		58.44	S7653-1kg	Sigma
KCl	2.5	1 mL	**2 mL**	**(2.5 M stock)**	74.55	P9333-500g	Sigma
$NaHCO_3$	26	2.1843	**4.3685**		84.01	S6297-250g	Sigma
NaH_2PO_4	1	0.8 mL	**1.6 mL**	**(1.25 M stock)**	119.98	S8282-500g	Sigma
D-Glucose	10	1.8016	**3.6032**		180.16	G7528-250g	Sigma
$CaCl_2$	2	2 mL	**4 mL**	**(1 M stock)**	110.98	21114-1L	Honeywell Fluke
$MgCl_2$	1	1 mL	**2 mL**	**(1 M stock)**	95.21	63020-1L	Honeywell Fluke
Ascorbic acid	*2*	*0.3522*	***0.7045***		*176.12*	*A92902-100G*	*Sigma*
Sodium pyruvate	*2*	*0.2201*	***0.4402***		*110.04*	*P2256-100G*	*Sigma*
Myo-inositol	*3*	*0.5405*	***1.0810***		*180.16*	*I5125-500G*	*Sigma*

Always start with ultrapure ddH_2O amounting to ~80% of the target volume.
Add all ingredients except for the divalents (Ca^{2+} and Mg^{2+}).
Adjust pH to 7.3-7.4 with NaOH or HCl if necessary. Once the pH is in range, add Ca^{2+} and Mg^{2+}.
Top off the volume with ultrapure ddH_2O.

Check osmolality: ~305 mOsm/kg (if adding the components in italics, otherwise ~295 mOsm/kg).
Ascorbic acid is a free radical scavenger.
Sodium pyruvate and Myo-inositol are additional energy sources that help cellular metabolism.

Prior to use, equilibrate the solution with 95% O_2, 5% CO_2 for at least 30 min.
Adjust pH again right before use if necessary.

Figure S2. Recipe for Recording ACSF.

2.2 [PATH B - Perfusion with ACSF under terminal isoflurane anaesthesia]

Path B follows the second of two alternative slicing methods. Briefly, it consists fn intracardially perfusing the mouse with ice-cold slicing ACSF under terminal isoflurane anaesthesia, followed by decapitation, brain extraction, and slicing in ice-cold slicing ACSF, incubation in slicing ACSF for 10-12 min at 32°C, followed by incubation in holding ACSF for 1 h at room temperature, and finally transferral of a slice to the recording chamber perfused with recording ACSF at 32-34°C or at room temperature.

In general, preparing 2 L of the slicing ACSF, 1 L of the holding ACSF, and 2 L of the recording ACSF should be enough for four days of experiments. Once prepared, store the solutions in the fridge at 4°C and try not to use them if older than a week. Make sure to use ultrapure ddH2O and to sterilise all glassware by baking for 2 hours at 200°C the previous day.

Citation: Oriol Pavon Arocas, Tiago Branco Preparation of acute midbrain slices containing the superior colliculus and periaqueductal gray for patch-clamp recordings https://dx.doi.org/10.17504/protocols.io.8epv51pz5l1b/v6

This is an open access protocol distributed under the terms of the **Creative Commons Attribution License** (https://creativecommons.org/licenses/by/4.0/), which permits unrestricted use, distribution, and reproduction in any medium,

Slicing ACSF (NMDG-HEPES)

For each experiment you will need ~500 mL of solution: ~150 mL for the first recovery chamber and ~350 mL to make the slush used for the intracardial perfusion and slicing steps (see Section 3 for more details).

- Slicing ACSF (in mM): 96 NMDG, 2.5 KCl, 25 NaHCO3, 1.25 NaH2PO4, 20 HEPES, 25 D-glucose, 5 sodium L-ascorbate, 2 thiourea, 3 sodium pyruvate, 3 myo-inositol, 12 N-acetyl-L-cysteine, 0.01 taurine, 0.5 CaCl2, 10 magnesium sulfate, with an osmolality of ~300 mOsm/kg.
- Add HCl to bring pH in range before adding the CaCl2 and MgSO4.
- If you need to reduce/increase osmolality, reduce/increase the amount of NMDG (and adjust pH accordingly) to avoid modifying the other ion concentrations.

Citation: Oriol Pavon Arocas, Tiago Branco Preparation of acute midbrain slices containing the superior colliculus and periaqueductal gray for patch-clamp recordings https://dx.doi.org/10.17504/protocols.io.8epv51pz5l1b/v6

This is an open access protocol distributed under the terms of the **Creative Commons Attribution License** (https://creativecommons.org/licenses/by/4.0/), which permits unrestricted use, distribution, and reproduction in any medium,

Path B - Slicing and recovery ACSF (NMDG-HEPES)						
for intracardial perfusion (20-50 mL of ice-cold solution) and tissue slicing						
recovery period <15 min at 32-34°C (less time if using <6 week old mice)						
excellent for mouse ages from 5 weeks to 1 year old or older						
	**mM**	**MW**	**g/2L**	**g/L**	**g/500 mL**	
NMDG	96	195.21	**37.4803**	18.7402	9.3701	
HCl (see notes)	*96*		*Add before $MgSO_4$ & $CaCl_2$*			**(5 M stock)**
KCl	2.5	74.55	**2 mL**	1 mL	0.5 mL	**(2.5 M stock)**
$NaHCO_3$	25	84.01	**4.2005**	2.1003	1.0501	
NaH_2PO_4	1.25	119.98	**2 mL**	1 mL	0.5 mL	**(1.25 M stock)**
HEPES	20	238.30	**9.5320**	4.7660	2.3830	
D-Glucose	25	180.16	**9.0080**	4.5040	2.2520	
Sodium L-ascorbate	5	198.11	**1.9811**	0.9906	0.4953	
Thiourea	2	76.12	**0.3045**	0.1522	0.0761	
Sodium pyruvate	3	110.04	**0.6602**	0.3301	0.1651	
Myo-inositol	3	180.16	**1.0810**	0.5405	0.2702	
N-acetyl-L-cysteine	12	163.19	**3.9166**	1.9583	0.9791	
Taurine	0.01	125.15	**2 mL**	1 mL	0.5 mL	**(10 mM stock)**
$CaCl_2$	0.5	110.98	**1 mL**	0.5 mL	250 µL	**(1 M stock)**
$MgSO_4.7H_2O$	10	246.47	**20 mL**	10 mL	5 mL	**(1 M stock)**

Always start with ultrapure ddH_2O amounting to ~80% of the target volume.
Add all ingredients except for the divalents (Ca^{2+} and Mg^{2+}) and HCl.
pH will be ~10 as NMDG is highly basic.
Adjust pH to 7.3-7.4 with ~15 mL of 5 M HCl for 1 L.
Once the pH is in range, add Ca^{2+} and Mg^{2+}.
Top off the volume with ultrapure ddH_2O.

Check osmolality: ~295-305 mOsm/kg. Adjust with water or NMDG powder.
Check pH again and adjust if necessary.

Prior to use, equilibrate the solution with 95% O_2, 5% CO_2 for at least 30 min.
Adjust pH to ~7.4 with NMDG powder on the day of use (gets acidic when cold).

Figure S3. Recipe for Slicing and recovery ACSF (NMDG-HEPES).

Holding ACSF (HEPES)

For each experiment you will need ~150 mL of solution for the second recovery chamber.

- Holding ACSF (in mM): 87 NaCl, 2.5 KCl, 25 NaHCO3, 1.25 NaH2PO4, 20 HEPES, 25 D-glucose, 5 sodium L-ascorbate, 2 thiourea, 3 sodium pyruvate, 3 myo-inositol, 12 N-acetyl-L-cysteine, 0.01 taurine, 2 CaCl2, 2 magnesium sulfate, with an osmolality of ~300 mOsm/kg.
- Add NaOH to bring pH in range before adding the CaCl2 and MgSO4.

Citation: Oriol Pavon Arocas, Tiago Branco Preparation of acute midbrain slices containing the superior colliculus and periaqueductal gray for patch-clamp recordings https://dx.doi.org/10.17504/protocols.io.8epv51pz5l1b/v6

This is an open access protocol distributed under the terms of the **Creative Commons Attribution License** (https://creativecommons.org/licenses/by/4.0/), which permits unrestricted use, distribution, and reproduction in any medium,

Path B - Holding ACSF (HEPES)						
for tissue maintenance at room temperature prior to recording						
transfer slices with minimum NMDG-Cl solution carryover						
	**mM**	**MW**	**g/2L**	**g/L**	**g/500 mL**	
NaCl	87	58.44	10.1686	**5.0843**	2.5421	
KCl	2.5	74.55	2 mL	**1 mL**	0.5 mL	**(2.5 M stock)**
$NaHCO_3$	25	84.01	4.2005	**2.1003**	1.0501	
NaH_2PO_4	1.25	119.98	2 mL	**1 mL**	0.5 mL	**(1.25 M stock)**
HEPES	20	238.30	9.5320	**4.7660**	2.3830	
D-Glucose	25	180.16	9.0080	**4.5040**	2.2520	
Sodium L-ascorbate	5	198.00	1.9800	**0.9900**	0.4950	
Thiourea	2	76.12	0.3045	**0.1522**	0.0761	
Sodium pyruvate	3	110.04	0.6602	**0.3301**	0.1651	
Myo-inositol	3	180.16	1.0810	**0.5405**	0.2702	
N-acetyl-L-cysteine	12	163.19	3.9166	**1.9583**	0.9791	
Taurine	0.01	125.15	2 mL	**1 mL**	0.5 mL	**(10 mM stock)**
$CaCl_2$	2	110.98	4 mL	**2 mL**	1 mL	**(1 M stock)**
$MgSO_4.7H_2O$	2	246.47	4 mL	**2 mL**	1 mL	**(1 M stock)**

Always start with ultrapure ddH_2O amounting to ~80% of the target volume.
Add all ingredients except for the divalents (Ca^{2+} and Mg^{2+}).
pH will be ~6.2.
Adjust pH to 7.3-7.4 with ~3.4 mL of 5 M NaOH to 1 L.
Once the pH is in range, add Ca^{2+} and Mg^{2+}.
Top off the volume with ultrapure ddH_2O.

Check osmolality: ~295-305 mOsm/kg. Adjust with water.
Adjust pH to 7.3-7.4 with NaOH or HCl if necessary.

Prior to use, equilibrate the solution with 95% O_2, 5% CO_2 for at least 30 min.
Adjust pH again on the day of use if necessary.

Figure S4. Recipe for Holding ACSF (HEPES).

Recording ACSF

For each experiment you will need ~250 mL of solution for superfusing the slices during recording. This is given as an approximation under the assumption that you recirculate the solution and do not need to wash in specific compounds. If you do not recirculate the solution or need to test the effect of washing in a specific compound, you will need more solution for each experiment.

Below are the recipes for two alternative recording solutions, which can be further modified to fulfill the specific needs of your tissue of interest. The first solution (Figure S5) is the one described in Gouwens, Sorensen, Berg, et al., 2019 and in Gouwens, Sorensen, et al., 2020 (for a full list of references see Section 8.11).

- Recording ACSF (in mM): 126 NaCl, 2.5 KCl, 26 NaHCO3, 1.25 NaH2PO4, 12.5

Citation: Oriol Pavon Arocas, Tiago Branco Preparation of acute midbrain slices containing the superior colliculus and periaqueductal gray for patch-clamp recordings https://dx.doi.org/10.17504/protocols.io.8epv51pz5l1b/v6

This is an open access protocol distributed under the terms of the **Creative Commons Attribution License** (https://creativecommons.org/licenses/by/4.0/), which permits unrestricted use, distribution, and reproduction in any medium,

D-glucose, 2 CaCl2, and 1 magnesium sulfate (equilibrated with 95% O2 and 5% CO2), with an osmolality of ~300 mOsm/kg.

- Equilibrate with 95% O2 and 5% CO2 and adjust pH to 7.3-7.4 before adding the CaCl2 and MgSO4.

Path B - Recording ACSF (standard)

for recording at 32-34°C or at room temperature

	mM	MW	g/2L	g/L	g/500mL	
NaCl	126	58.44	**14.7269**	7.3634	3.6817	
KCl	2.5	74.55	**2 mL**	1 mL	0.5 mL	**(2.5 M stock)**
$NaHCO_3$	26	84.01	**4.3685**	2.1843	1.0921	
NaH_2PO_4	1.25	119.98	**2 mL**	1 mL	0.5 mL	**(1.25 M stock)**
D-Glucose	12.5	180.16	**4.5040**	2.2520	1.1260	
$CaCl_2$	2	110.98	**4 mL**	2 mL	1 mL	**(1 M stock)**
$MgSO_4.7H_2O$	1	246.47	**2 mL**	1 mL	0.5 mL	**(1 M stock)**

Always start with ultrapure ddH_2O amounting to ~80% of the target volume.
Add all ingredients except for the divalents (Ca^{2+} and Mg^{2+}).
Adjust pH to 7.3-7.4 with NaOH or HCl if necessary. Once in range, add Ca^{2+} and Mg^{2+}.
Top off the volume with ultrapure ddH_2O.

Check osmolality: ~295-305 mOsm/kg.

Prior to use, equilibrate the solution with 95% O_2, 5% CO_2 for at least 30 min.
Adjust pH again right before use if necessary.

Figure S5. Recipe for Recording ACSF (standard).

The second solution (Figure S6) is a mix between the recording ACSF from Path A and the one above. It has essentially been modified to contain HEPES (which reduces edema and increases pH buffering capacity in the physiological range), ascorbic acid (to reduce oxidative stress), and sodium pyruvate and myo-inositol (to provide additional entry points into cellular metabolism).

- Recording ACSF (in mM): 126 NaCl, 2.5 KCl, 26 NaHCO3, 1.25 NaH2PO4, 12.5 D-glucose, 2 CaCl2, and 1 magnesium sulfate (equilibrated with 95% O2 and 5% CO2), with an osmolality of ~300 mOsm/kg.
- Optionally, you can add (in mM): 5 HEPES, 2 ascorbic acid, 2 sodium pyruvate, and 3 myo-inositol. If added, osmolality will increase by ~10 mOsm/kg.
- Equilibrate with 95% O2 and 5% CO2 and adjust pH to 7.3-7.4 before adding the CaCl2 and MgSO4.

Citation: Oriol Pavon Arocas, Tiago Branco Preparation of acute midbrain slices containing the superior colliculus and periaqueductal gray for patch-clamp recordings https://dx.doi.org/10.17504/protocols.io.8epv51pz5l1b/v6

This is an open access protocol distributed under the terms of the **Creative Commons Attribution License** (https://creativecommons.org/licenses/by/4.0/), which permits unrestricted use, distribution, and reproduction in any medium,

Path B - Recording ACSF (modified)

for recording at 32-34°C or at room temperature

	mM	MW	g/2L	g/L	g/500mL	
NaCl	125	58.44	**14.6100**	7.3050	3.6525	
KCl	2.5	74.55	**2 mL**	1 mL	0.5 mL	**(2.5 M stock)**
$NaHCO_3$	26	84.01	**4.3685**	2.1843	1.0921	
NaH_2PO_4	1.25	119.98	**2 mL**	1 mL	0.5 mL	**(1.25 M stock)**
HEPES	*5*	*238.30*	***2.3830***	*1.1915*	*0.5958*	
D-Glucose	10	180.16	**3.6032**	1.8016	0.9008	
$CaCl_2$	2	110.98	**4 mL**	2 mL	1 mL	**(1 M stock)**
$MgSO_4.7H_2O$	1	246.47	**2 mL**	1 mL	0.5 mL	**(1 M stock)**
Ascorbic acid	*2*	*176.12*	***0.7045***	*0.3522*	*0.1761*	
Sodium pyruvate	*2*	*110.04*	***0.4402***	*0.2201*	*0.1100*	
Myo-inositol	*3*	*180.16*	***1.0810***	*0.5405*	*0.2702*	

Always start with ultrapure ddH_2O amounting to ~80% of the target volume.
Add all ingredients except for the divalents (Ca^{2+} and Mg^{2+}).
Adjust pH to 7.3-7.4 with NaOH or HCl if necessary. Once in range, add Ca^{2+} and Mg^{2+}.
Top off the volume with ultrapure ddH_2O.

Check osmolality: ~295-305 mOsm/kg.
HEPES reduces post-slicing edema and tissue swelling.
Ascorbic acid is a free radical scavenger.
Sodium pyruvate and Myo-inositol are additional energy sources that help cellular metabolism.

Prior to use, equilibrate the solution with 95% O_2, 5% CO_2 for at least 30 min.
Adjust pH again right before use if necessary.

Figure S6. Recipe for Recording ACSF (modified).

2.3 [General considerations]

- If possible, set up a dedicated clean area and have a specific set of tools for slice work, including for perfusing the animals and extracting the brain. Make sure to avoid any contamination with PFA.
- If possible, use a specific set of glassware for slice electrophysiology experiments and wash it thoroughly with ddH20 independently from the rest of laboratory glassware.
- Sterilise the glassware used for preparing and storing solutions and for incubating slices: bake at 200°C for 2 h to minimise the risk of any bacterial growth. Do this at the end of each experiment after thoroughly washing with ddH2O to remove any salts residues.
- Always check the pH and osmolality of the stock solutions and keep a log for record keeping.
- Make sure to vigorously shake and mix the stock solutions before taking the

Citation: Oriol Pavon Arocas, Tiago Branco Preparation of acute midbrain slices containing the superior colliculus and periaqueductal gray for patch-clamp recordings https://dx.doi.org/10.17504/protocols.io.8epv51pz5l1b/v6

This is an open access protocol distributed under the terms of the **Creative Commons Attribution License** (https://creativecommons.org/licenses/by/4.0/), which permits unrestricted use, distribution, and reproduction in any medium,

amount needed for the day.

Preparation of tools, instruments, and solutions for the day 1h

3

This section outlines the preparations that need to be in place before starting either slicing procedure. These include how to get the solutions needed for the day ready for use, the tools and instruments that will be used, how to set up the vibratome and perform blade alignment, and how to prepare the slush out of the slicing ACSF.

3.1 [Get solutions ready]

- Assemble two recovery chambers: for each chamber use a 150 mL glass beaker, a double-threaded plastic cap (or a 3D printed alternative as the one available here), some sterile dressing, and a 2 mL syringe from which the tip has been cut and a needle inserted at the bottom to make a support for the bubblestone. Ideally the chamber should be disassembled after every use to thoroughly clean all parts with ddH2O and to bake the glass beaker to sterilise it.

Depending on the path you follow, do:

- **[PATH A - Decapitation under terminal isoflurane anaesthesia]:** fill one recovery chamber with ~150 mL of slicing ACSF (sucrose) and the other with ~150 mL of recording ACSF. Place both beakers in the heating blocks (or water bath) so the solutions reach 35°C (Figure S7). Fill the large beaker with ~350 mL of slicing ACSF (sucrose) and leave on ice (Figure S8). Turn carbogen on and bubble all solutions for at least 20-30 min - this will ensure the pH is equilibrated and the solutions are oxygenated and at the right temperature.
- **[PATH B - Perfusion with ACSF under terminal isoflurane anaesthesia]:** fill one recovery chamber with ~150 mL of slicing ASCF (NMDG-HEPES) and the other with ~150 mL of holding ACSF (HEPES). Place both beakers in the heating blocks (or water bath) so the solutions reach 32°C (Figure S7). Fill the large beaker with ~350 mL of slicing ACSF (NMDG-HEPES) and leave on ice (Figure S8). Turn carbogen on and bubble both solutions for at least 20-30 min - this will ensure the pH is equilibrated and the solutions are oxygenated and at the right temperature. Importantly, remove the recovery chamber containing holding ACSF from the heating block before you start the slicing procedure - this solution needs to be at room temperature and can be momentarily placed on the heating block to make sure it reaches room temperature quick enough (otherwise it may take longer to go from 4°C to room temperature).

Citation: Oriol Pavon Arocas, Tiago Branco Preparation of acute midbrain slices containing the superior colliculus and periaqueductal gray for patch-clamp recordings https://dx.doi.org/10.17504/protocols.io.8epv51pz5l1b/v6

This is an open access protocol distributed under the terms of the **Creative Commons Attribution License** (https://creativecommons.org/licenses/by/4.0/), which permits unrestricted use, distribution, and reproduction in any medium,

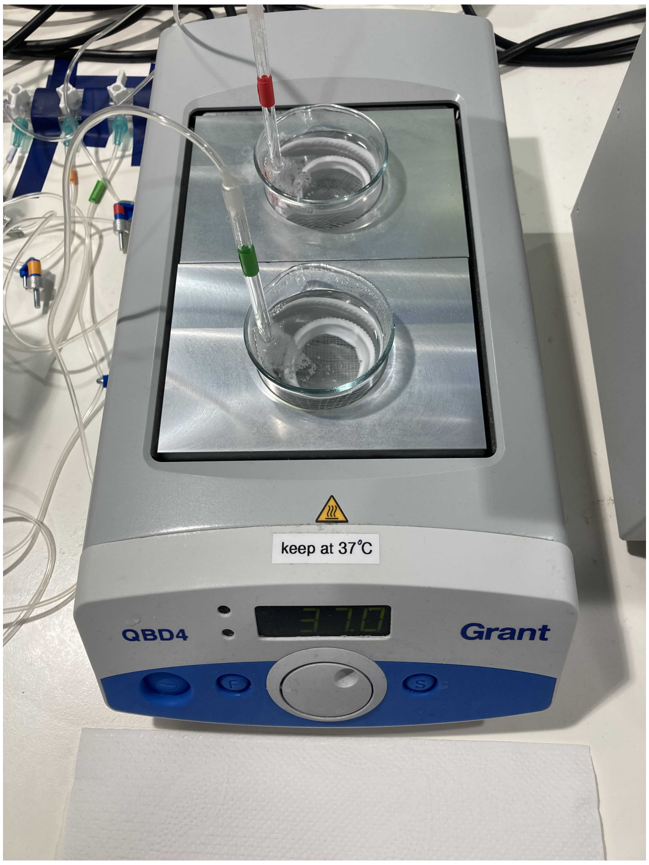

Figure S7. Recovery chambers in heating blocks.

Make sure to remove bubbles from the net of the recovery chambers (bubbles right underneath a slice will prevent the solution from evenly reaching the tissue). Cover all the beakers with parafilm to prevent evaporation (otherwise the osmolality will change). Check the pH and osmolality once the solutions have been bubbling for 20-30 min and have reached the desired temperature. Adjust if necessary.

Note that the exact temperature settings will depend on the specific heating system - if you can, do validate the temperature of the solution with an external thermometer. For the heating blocks listed in the materials section of this protocol (Figure S7), the temperature was set to 37°C (or 34°C for Path B) as this ensured the solutions reached 35°C (or 32°C for Path B).

3.2 [Prepare tools for slicing]

- Scissors for decapitation: Student Surgical Scissors Straight Sharp-Blunt (14.5 cm).
- Fine scissors for skin: Extra Fine Bonn Scissors Straight (8.5 cm).
- Small serrated scissors for skull: Vannas Tübingen Spring Scissors Straight

Citation: Oriol Pavon Arocas, Tiago Branco Preparation of acute midbrain slices containing the superior colliculus and periaqueductal gray for patch-clamp recordings https://dx.doi.org/10.17504/protocols.io.8epv51pz5l1b/v6

This is an open access protocol distributed under the terms of the **Creative Commons Attribution License** (https://creativecommons.org/licenses/by/4.0/), which permits unrestricted use, distribution, and reproduction in any medium,

Serrated (8.5 cm).
- Fine tweezers to detach meninges from brain: Dumont #5 Fine Forceps Biology Inox.
- Forceps to separate skull: Micro-Adson Forceps Serrated.
- Blade to cut frontal part of the brain and cerebellum: Scalpel Handle #4 and sterile blade.
- Spatula to remove brain from skull: Probe and Spatula (14 cm).
- Spoon to immerse brain in ACSF slush: Moria MC17 Perforated Spoon (20 mm tip diameter).
- Brush to move and reposition the slices: Major Brushes size 2.
- Superglue (Universal) to glue the brain on the magnetised block of the vibratome. Make sure it has not dried out.
- Syringe and needle to separate and mark cut slices from brain: 1 mL syringe with a stainless steel needle (e.g. brown - BD Microlance 3, 0.45x13 mm). Can be carefully washed and reused.
- Glass pipette to make a slice collector to pick and transport brain slices.
- Filter paper on a glass petri dish.
- Crystallizing dish without spout (150 mL) to perform the brain extraction (so that you can keep immersing the brain into cold solution at every step).
- Small 50 mL or medium 150 mL beaker to deposit the head after decapitation.
- Two medium 150 mL beakers for the recovery chambers.
- Big 400 mL beaker for the slicing ACSF.
- Falcon tube (50 mL).
- Ice pan full of ice.
- Double edge blades for vibratome (Personna).
- Cotton buds and Acetone to clean the blades before slicing.
- Tissue paper.
- Magnetised block from vibratome (where the brain will be glued before slicing).

[!] An additional thing you can try is to keep the tip of the tools on ice so they are as cold as possible during the brain extraction. This will help reduce the temperature of the tissue while extracting the brain.

[Prepare slicing chamber]

- Assemble the vibratome support and the slicing chamber and add some ice around it (see bottom left of Figure S8).
- Store in the freezer until needed to ensure it is as cold as possible.
- Leave the circular magnetised block out to prevent condensation when taking it out of the freezer as this can stop the glue/brain from sticking on to it.

Citation: Oriol Pavon Arocas, Tiago Branco Preparation of acute midbrain slices containing the superior colliculus and periaqueductal gray for patch-clamp recordings https://dx.doi.org/10.17504/protocols.io.8epv51pz5l1b/v6

This is an open access protocol distributed under the terms of the **Creative Commons Attribution License** (https://creativecommons.org/licenses/by/4.0/), which permits unrestricted use, distribution, and reproduction in any medium,

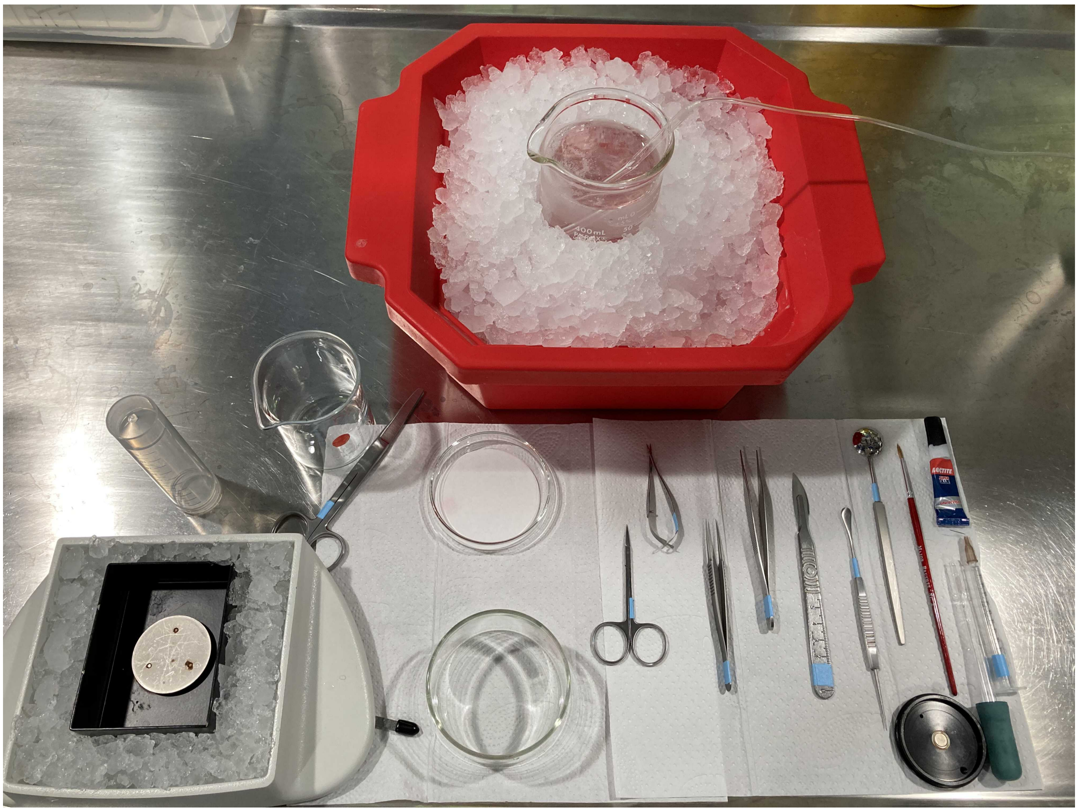

Figure S8. Tools for preparing acute midbrain slices from young adult mice.

3.3 [Vibratome set up and blade alignment - Leica VT1200S]

- Use a Personna blade to carefully clean the grease (without bending it!): dip a cotton bud in acetone and use it to gently wipe away any dirt or grease on both sides of the blade. Then briefly rinse with 70% ethanol and then thoroughly clean with ddH20. Dry it before mounting.
- It is possible to break the blade in half to use the second half the following day - we tend not to do that to minimise any unwanted bending of the blade.
- Place the blade on the vibratome and tighten. Do not lower it yet.
- Mount and connect the Vibrocheck. Then lower the blade to an horizontal position and follow the vibrocheck alignment procedure to align the blade (Figure S9). It is very important to do this with every new blade.
- Once it is aligned, keep it running for a few minutes to make sure it doesn't change with time. Readjust if necessary.
- Exit the vibrocheck mode, move the blade holder up, and remove the vibrocheck and cable.
- The vibratome is now ready for use.

Citation: Oriol Pavon Arocas, Tiago Branco Preparation of acute midbrain slices containing the superior colliculus and periaqueductal gray for patch-clamp recordings https://dx.doi.org/10.17504/protocols.io.8epv51pz5l1b/v6

This is an open access protocol distributed under the terms of the **Creative Commons Attribution License** (https://creativecommons.org/licenses/by/4.0/), which permits unrestricted use, distribution, and reproduction in any medium,

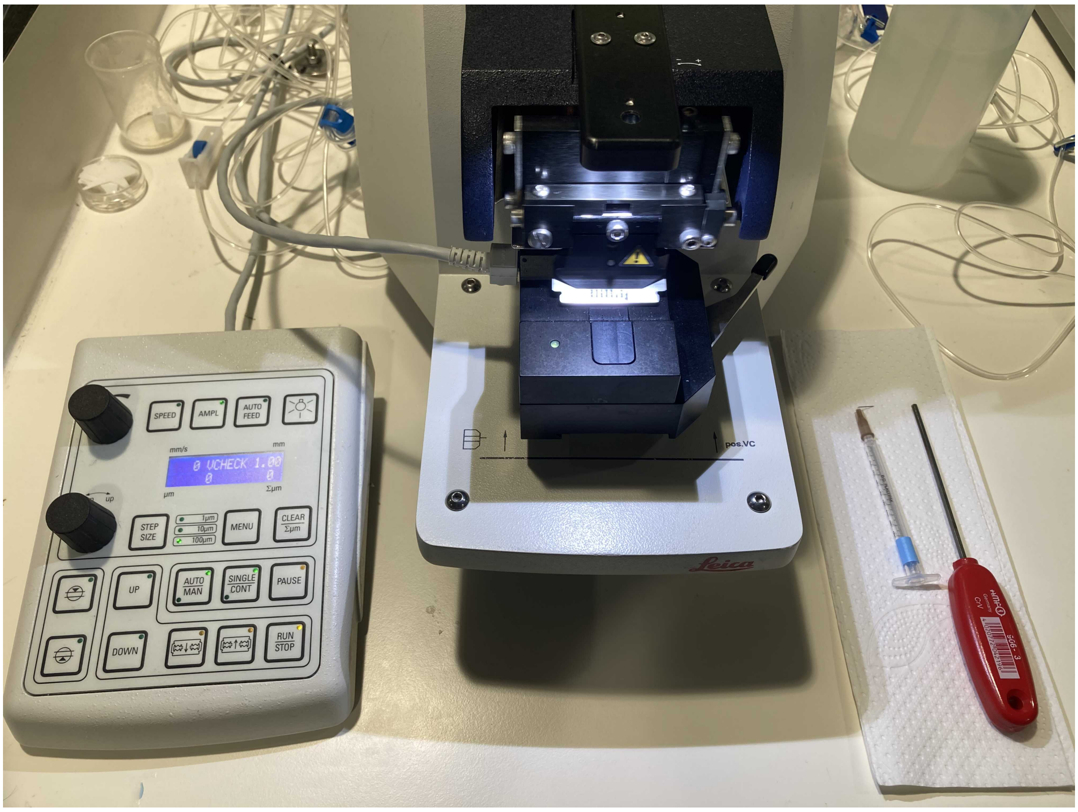

Figure S9. Blade alignment with the Vibrocheck on a Leica VT1200S.

3.4 [Prepare slush of slicing solution]

In order to bring the slicing solution as close to 0°C as possible, an Ice-Cream Maker can be used to prepare a slush.

- Make sure that the beaker with ~350 mL of slicing ACSF has been bubbling for at least 20-30 min on ice (Figure S8).
- Before proceeding to make the slush, check the pH of the solution (pH is temperature dependent and will probably have changed, usually by turning acidic). Adjust with either NaOH/NaHCO3 (if using the sucrose ACSF) or NMDG (if using the NMDG-HEPES ACSF). Once the pH is in range, proceed to the next step.
- Remove the Ice-Cream Maker from the freezer.
- Pour the ~350 mL of bubbled slicing ACSF in and continue to bubble (Figure S10).
- Use a 50 mL falcon tube to scrape off the ice that generates in the walls. Mix and repeat until you have a uniform slush (you don't want too much ice nor too much liquid).
- Pour the slush of slicing ACSF back to the 400 mL beaker.
- If available, use a clean blender on the slush to remove any sharp edges in the ice.
- Put the beaker with the slush on ice and continue to bubble with carbogen until

Citation: Oriol Pavon Arocas, Tiago Branco Preparation of acute midbrain slices containing the superior colliculus and periaqueductal gray for patch-clamp recordings https://dx.doi.org/10.17504/protocols.io.8epv51pz5l1b/v6

This is an open access protocol distributed under the terms of the **Creative Commons Attribution License** (https://creativecommons.org/licenses/by/4.0/), which permits unrestricted use, distribution, and reproduction in any medium,

used.

- Clean the Ice-Cream Maker with ddH2O and dry it quickly with a tissue (if not done quickly enough some paper will get stuck to the cold walls and it will need to be cleaned again). Put back in the freezer until next use.

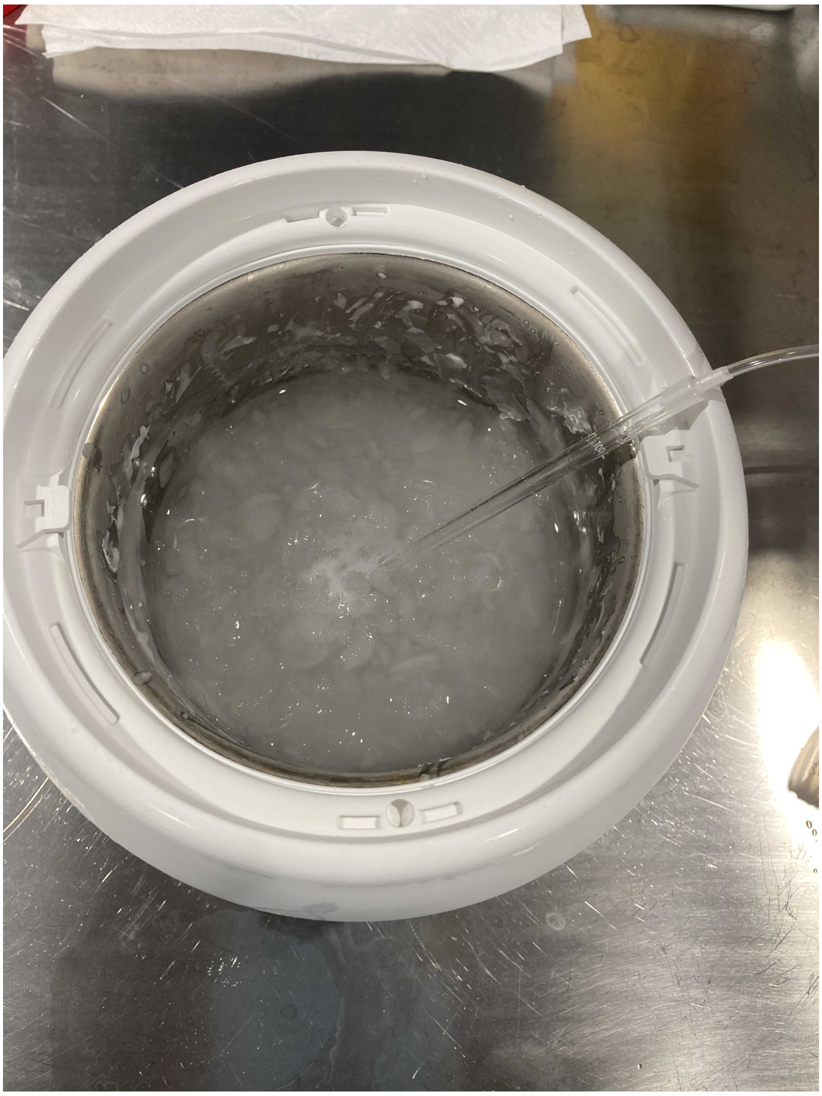

Figure S10. Preparation of a slush of slicing ACSF with an Ice-Cream Maker.

3.5 [Hood and anaesthetic chamber]

- Place clean tissue paper on the floor of the anaesthetic chamber.
- Place a disposal bag in the large glass beaker (to dispose of the carcass).
- For **PATH A - Decapitation under terminal isoflurane anaesthesia,** make sure you bring the small beaker with the slush made of the slicing ACSF (sucrose) and the scissors for decapitation (Figure S11).
- For **PATH B - Perfusion with ACSF under terminal isoflurane anaesthesia**, make sure you prepare a 20 mL syringe, four needles (to pin the paws), and the tray and silicon mat (where the animal will rest once

Citation: Oriol Pavon Arocas, Tiago Branco Preparation of acute midbrain slices containing the superior colliculus and periaqueductal gray for patch-clamp recordings https://dx.doi.org/10.17504/protocols.io.8epv51pz5l1b/v6

This is an open access protocol distributed under the terms of the **Creative Commons Attribution License** (https://creativecommons.org/licenses/by/4.0/), which permits unrestricted use, distribution, and reproduction in any medium,

anaesthetised). In addition, bring the small beaker with the slush made of the slicing ACSF (NMDG-HEPES) and load the syringe with it. Get the scissors for decapitation, a pair of forceps, and medium-size scissors to carry out the dissection to reach the heart. To keep the tray as cold as possible, you can add ice around the silicon mat.

- If working under a ventilated hood, switch the ventilation ON before opening the animal cage. Switch the scavenging system and the O2 inflow ON before you open the anaesthetic flow.

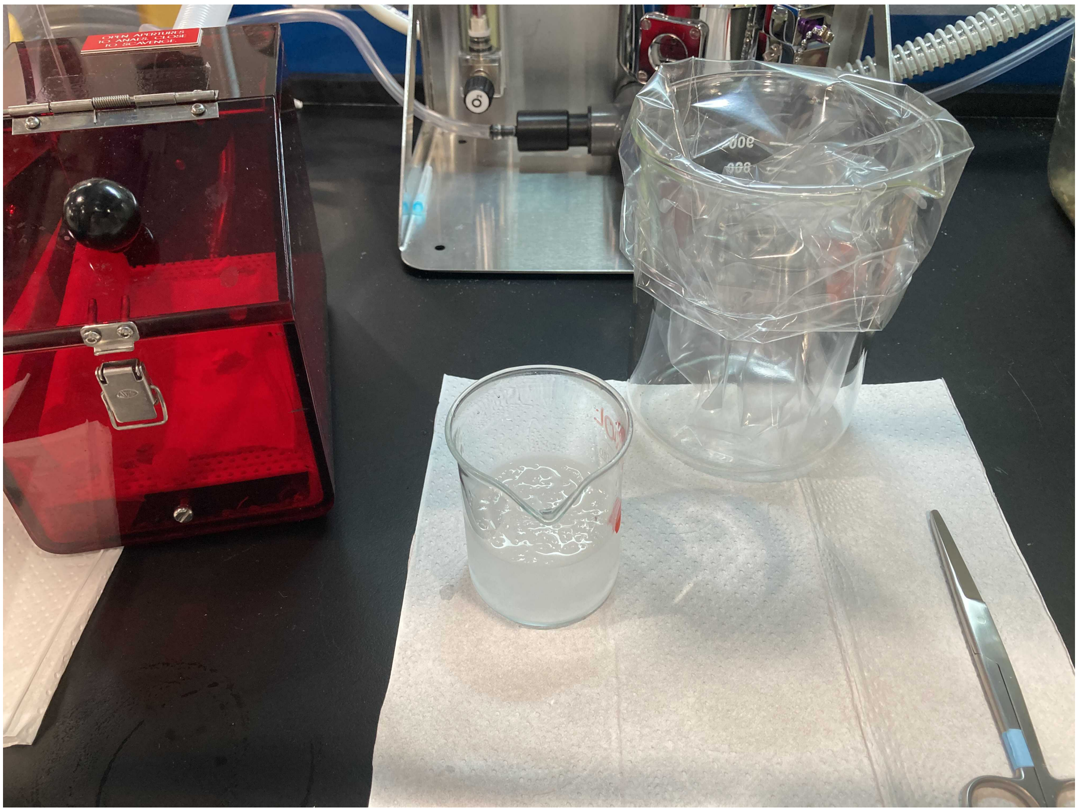

Figure S11. Anaesthetic chamber together with tools for decapitation under terminal anaesthesia.

3.6 [Electrophysiology rig]

- Fill a glass bottle with ~250 mL of recording ACSF.
- Turn the carbogen ON and leave the recording ACSF bubbling until use so it reaches room temperature before you start recording.
- Cover with parafilm to minimise loss of solution.

Brain extraction 10m

4

This section describes two different procedures: decapitation under terminal isoflurane anaesthesia and intracardial perfusion with ACSF under terminal isoflurane anaesthesia.

Citation: Oriol Pavon Arocas, Tiago Branco Preparation of acute midbrain slices containing the superior colliculus and periaqueductal gray for patch-clamp recordings https://dx.doi.org/10.17504/protocols.io.8epv51pz5l1b/v6

This is an open access protocol distributed under the terms of the **Creative Commons Attribution License** (https://creativecommons.org/licenses/by/4.0/), which permits unrestricted use, distribution, and reproduction in any medium,

Both procedures are followed by brain extraction and allow the preparation of coronal slices. The choice of method and ACSF recipe will very likely differ across brain regions, and you may need to make slight modifications to reach the optimal version for your particular experiment. Some suggestions in that direction can be found in Section 8.

It is very important to plan ahead - not only do you need to prepare solutions (and foresee any shortages in salts, water, or any other consumable you need) in advance, but you also need to ensure you have the right animals (genotype wise) at the most appropriate age for your experiment. Although the considerations and specific directives with regards to experimental animals will vary across institutions and will depend on experimental design and available equipment, the following sections provide a detailed account of how we executed each step. We provide this highly specific details in the hope that someone wishing to implement this method from scratch will have an example of how each step was successfully carried out in another institution.

[Common for Path A and Path B - Get the experimental subject]

- Follow the guidelines in place at the home institution to source experimental animals. If they need to be transferred from a separate breeding room or requested from an external company, do so with sufficient anticipation.
- If you require your animals to be in a particular age bracket for your experiment, plan accordingly. Note that the older the animal is, the more challenging the slicing procedure becomes and the more difficult it is to obtain good quality slices.
- Once you are ready to begin your experiment, fetch the cage and bring it to the area where you will prepare the slices. If possible, place under a ventilation hood and ensure you only open the cage with the ventilation ON.
- Make sure everything you need is ready before proceeding to anaesthetise the animal and begin the slicing procedure. This includes regularly checking the weight of the scavenging filter (replace it with a new one if the limit is exceeded and properly dispose of the old one) as well as the levels of isoflurane in the anaesthetic system (top it up if running low).

4.1 **[PATH A - Decapitation under terminal isoflurane anaesthesia]**

- Wear appropriate PPE: coat, mask, and gloves.
- Put a disposal bag in the glass beaker if you haven't done so already.
- Pour some slush of slicing ACSF in the small 150 mL beaker. Get the beaker and the scissors for decapitation to the area where you will perform the procedure.
- Switch ON the oxygen flow from the pipe in the wall, and also turn ON the oxygen flow in the anaesthesia system to around 2 mL/min.
- Switch the scavenging system ON and double check a clean tissue paper is present on the floor of the anaesthetic chamber.
- Turn the valve behind the anaesthetic chamber so that it is on the "scavenging" position before opening the isoflurane flow (the scavenging system is there to protect you from any isoflurane leaks).
- Proceed to anaesthetise the mouse: open the animal cage and gently cup the mouse of your choice (we usually have ear marks to identify them when group

Citation: Oriol Pavon Arocas, Tiago Branco Preparation of acute midbrain slices containing the superior colliculus and periaqueductal gray for patch-clamp recordings https://dx.doi.org/10.17504/protocols.io.8epv51pz5l1b/v6

This is an open access protocol distributed under the terms of the **Creative Commons Attribution License** (https://creativecommons.org/licenses/by/4.0/), which permits unrestricted use, distribution, and reproduction in any medium,

housed) and transfer to the anaesthetic chamber. If possible, avoid picking up by the tail as this leads to stress. Once the animal is in the anaesthetic chamber, switch the isoflurane pump ON by turning the wheel to 5 mL/min. Turn the valve behind the anaesthetic chamber to the "anaesthetise" position. While you wait for the isoflurane to have its effect, close the animal cage and move below the hood, so that the remaining animals can't see the rest of the procedure.
- Once the animal is under anaesthesia, open the chamber and check the reflexes by pinching the rear paw. If the anaesthesia levels are good, switch the isoflurane pump OFF and return the valve behind the anaesthetic chamber to "scavenging" position. Leave the scavenging system on for as long as you extract the brain.
- Take the animal out, hold over the beaker containing the slush of slicing ACSF, and place the large scissors around the neck. With a swift cut, proceed to perform the decapitation, making sure the head falls inside the ice-cold and already bubbled slush of slicing ACSF. Place the carcass in the bag of the large beaker.
- Quickly take the beaker to the vibratome area, making sure the head is completely immersed in the cold solution. The next steps are crucial and must be done as quickly as possible - ideally the time between decapitation and immersing the extracted brain in the slush of slicing ACSF should be less than 1 minute.

4.2 [PATH B - Perfusion with ACSF under terminal isoflurane anaesthesia]

- Wear appropriate PPE: coat, mask, and gloves.
- Put a disposal bag in the glass beaker if you haven't done so already.
- Pour some slush of slicing ACSF in the small 150 mL beaker. Get the beaker and the tools for dissection and decapitation (scissors for decapitation, scissors for skin, and forceps) to the area where you will perform the procedure.
- Load a 20 mL syringe with slush of slicing ACSF and mount a small brown needle on it. Press to squirt a bit of liquid and make sure there are no air bubbles in the syringe.
- Get the tray and the silicon mat together with four sterile small brown needles, which you will use to pin the animal. Place the anaesthetic mask on the silicon mat. Remove the cap from the needles and stick them into the silicon mat.
- Place the tools, syringe, and beaker on a piece of clean tissue paper next to the tray, ready for use.
- Switch ON the oxygen flow from the pipe in the wall, and also turn ON the flow in the anaesthesia system to around 2 mL/min.
- Switch the scavenging system ON and double check a clean tissue paper is present on the floor of the anaesthetic chamber.
- Turn the valve behind the anaesthetic chamber so that it is on the "scavenging" position before opening the isoflurane flow (this system is there to protect you from any isoflurane leaks).
- Proceed to anaesthetise the mouse: open the animal cage and gently cup the mouse of your choice (we usually have ear marks to identify them when group

Citation: Oriol Pavon Arocas, Tiago Branco Preparation of acute midbrain slices containing the superior colliculus and periaqueductal gray for patch-clamp recordings https://dx.doi.org/10.17504/protocols.io.8epv51pz5l1b/v6

This is an open access protocol distributed under the terms of the **Creative Commons Attribution License** (https://creativecommons.org/licenses/by/4.0/), which permits unrestricted use, distribution, and reproduction in any medium,

housed) and transfer to the anaesthetic chamber. If possible, avoid picking up by the tail as this leads to stress. Once the animal is in the anaesthetic chamber, switch the isoflurane pump ON by turning the wheel to 5 mL/min. Switch the valve behind the anaesthetic chamber to the "anaesthetise" position. While you wait for the isoflurane to have its effect, close the animal cage and move below the hood, so that the remaining animals can't see the rest of the procedure.

- Once the animal is under anaesthesia, open the chamber and check the reflexes by pinching the rear paw. If the anaesthesia levels are good, turn the valve to change the anaesthesia circuit from the chamber to the mask and transfer the mouse to the silicon mat. Carefully place the mask around the snout, making sure it stays into position. Switch the valve to change the scavenging circuit from the chamber to the mask and turn the valve behind the anaesthetic chamber from "anaesthetise" to "scavenging".
- With the mask well placed and the mouse still under anaesthesia, check the reflexes again. If the anaesthesia levels are still good, proceed to pin the animal to the silicon mat with one needle in each paw.
- Take the scissors with your dominant hand and the forceps with the other and proceed to make an incision on the skin at the level of the abdomen. Use the scissors to gain access to the abdominal cavity, and proceed to dissect up towards the rib cage until you see the diaphragm. Once you cut the diaphragm, the animal stops breathing properly and starts dying, so you need to be fast (otherwise perfusing will not be much of an improvement compared to decapitation). Carefully cut the diaphragm and cut around the rib cage until you see the heart. Use the forceps to lift the cut rib cage so you have good access to the heart. Carefully cut the right atrium - blood should start coming out. Quickly leave the scissors and get the syringe with the ice-cold slicing ACSF (squirt a bit of solution to ensure there are no bubbles in the tip). Use the forceps to position the heart and insert the needle in the left ventricle. Slowly empty the syringe without stopping, until all the solution has been used. If the perfusion works well, the liver should turn white and clean ACSF should be coming out the cut atrium towards the end.
- If you have a steady pulse and don't need the second hand to keep the syringe in place while perfusing, use it to remove the mask from the snout and switch the isoflurane pump OFF (leaving the scavenging system on). Sometimes during the perfusion some ACSF comes out of the snout - removing the mask ensures the solution doesn't end up in it.
- Once the syringe is empty and the perfusion is complete, remove the needles pinning the animal to the silicon mat and take the big scissors. Carefully lift the mouse and hold over the beaker containing the remaining slush of slicing ACSF. Place the large scissors around the neck and, with a swift cut, proceed to perform the decapitation, making sure the head falls inside the ice-cold and already bubbled slush of slicing ACSF. Place the carcass in the bag of the large beaker.
- If you haven't done so yet, switch the isoflurane pump OFF and leave the scavenging system on for as long as you extract the brain. After that, make sure you come back to change the valves back to the chamber circuit and leave the scavenging on for a bit longer. Remember to close the oxygen flow

Citation: Oriol Pavon Arocas, Tiago Branco Preparation of acute midbrain slices containing the superior colliculus and periaqueductal gray for patch-clamp recordings https://dx.doi.org/10.17504/protocols.io.8epv51pz5l1b/v6

This is an open access protocol distributed under the terms of the **Creative Commons Attribution License** (https://creativecommons.org/licenses/by/4.0/), which permits unrestricted use, distribution, and reproduction in any medium,

and the scavenger when you are done.

- Quickly take the beaker to the vibratome area, making sure the head is completely immersed in the cold solution. The next steps are crucial and must be done as quickly as possible - ideally the time between decapitation and immersing the extracted brain in the slush of slicing ACSF should be less than 1 minute.

4.3 [Common for Path A and Path B - Brain extraction (<1 min)]

- Pour some slush from the 400 mL beaker on ice (where the slush of slicing ACSF has been bubbling all this time) into the crystallizing dish. This is where you will keep immersing the head while doing the brain extraction, in order to keep it as cold as possible. Having a separate beaker for the extraction and decapitation also reduces the amount of blood carried over.
- Take the head from the small beaker and place it in the crystallizing dish with clean slush.
- Hold the head to ensure the skull is stable by applying pressure to the snout (adopt a sort of pinching position with the thumb and index that surrounds the snout and with the maximal pressure on the phalanges - never apply pressure to the skull itself, as you will compress and damage the brain). Place your middle finger beneath the lower jaw as a support. Importantly, you should ensure you have practiced this in advance (for instance, with a carcass or a 3D printed model of a mouse skull) - having a good and stable grip will be critical to extract the brain as quickly and safely as possible.
- Use the medium size scissors to cut through the skin along the midline and expose the skull. Start at the back of the neck and make your way until the snout. Fully immerse the head in the ice-cold slush of slicing ACSF.
- With the brain still immersed in the slush, switch to the small serrated scissors. Start to very carefully cut through the bone from the foramen magnum in the following sequence.
- (1) The first two cuts will be to the sides, from the foramen magnum around the temporal bone until the zygomatic bone or the ear canal (surrounding the cerebellum). This two cuts (one to the right and one to the left) are very important as will reach the insertion of most meninges: making sure these are cut will prevent they slash through the brain and separate the cerebellum and inferior colliculus from the superior colliculus and periaqueductal gray.
- (2) Next, once again insert the small serrated scissors in the foramen magnum and proceed to cut the skull along the midline suture from caudal to rostral until you reach the snout. Make sure you cut "up" when you advance with the scissors (never "down" towards the brain) to avoid pressing the bone against the tissue. Fully immerse the head in the ice-cold slush of slicing ACSF.
- (3) Finally, make two small cuts from the midline to the laterals at the level of the olfactory bulb. Fully immerse the head in the ice-cold slush of slicing ACSF.
- **N.B**: although you must be fast, it is also necessary to be very careful and to keep mechanical stress to the minimum - the midbrain is incredibly sensitive. In addition, as already mentioned above, always make the scissor cuts in the upper direction, so that instead of applying pressure to the skull you are lifting it away from the brain. In many cases, insufficient slice quality is due to

Citation: Oriol Pavon Arocas, Tiago Branco Preparation of acute midbrain slices containing the superior colliculus and periaqueductal gray for patch-clamp recordings https://dx.doi.org/10.17504/protocols.io.8epv51pz5l1b/v6

This is an open access protocol distributed under the terms of the **Creative Commons Attribution License** (https://creativecommons.org/licenses/by/4.0/), which permits unrestricted use, distribution, and reproduction in any medium,

problems at this very early stage.

- With the brain still immersed in the slush, switch to the fine Dumont forceps. Very carefully lift each half of the skull and check if the dura mater is still stuck to the brain or if it instead lifts with the bone. If the dura is still stuck to the brain, make sure you carefully detach it by inserting the tip of the forceps right below it and moving the tip across the bone (from front to back). Do the same for both sides and make sure the meninges are out of the picture before proceeding. Fully immerse the head in the ice-cold slush of slicing ACSF. This is an essential step, if not done the remaining meninges can cut the entire brain when you lift the skull in the next step.
- With the brain still immersed in the slush, switch to the thick serrated forceps. Remove the skull by lifting the cut bone with the forceps while moving the hand that holds the head in the opposite direction. Do the same for the other side. Fully immerse the head in the ice-cold slush of slicing ACSF.
- With the brain still immersed in the slush, take the blade. Make sure you are holding the head as flat in the horizontal plane as possible. Make one cut through the brain at the level of the cerebellum and one in the front to remove a good chunk of the frontal lobe. It is very important that this second cut is as straight as possible in the vertical plane as this is the surface the brain will stand on when glued to the magnetised block.
- With the brain still immersed in the slush, grab the spatula and carefully scoop the cut parts out. Then carefully separate the useful part of the brain from the base of the skull and gently transfer (do NOT drop) to the 400 mL beaker with the remaining slush of slicing ACSF (which has been bubbling on ice until now). Use the perforated spoon to carefully scoop a pocket in the slush so that the brain remains completely submerged in it. Make sure the bubbling is not too close to the brain.
- Ideally, the brain brain removal steps should take less than a minute. You can leave the brain to recover for a minute or so in the cold solution, this will ensure to slow down the metabolism and reduce excitotoxicity.
- In the meantime, bring the now empty skull back to the hood and put it in the plastic bag together with the carcass. Properly dispose of the carcass. Close the oxygen flow from the wall and watch the rubber that indicates the flow rate fall to zero. Make sure the isoflurane pump is off. Then close the circuit from the anaesthetic chamber and switch off the scavenging system. Clean the area with ddH2O from any blood or ACSF and wipe with 70% ethanol. Switch the hood ventilation off. Bring the beaker and scissors to the sink. You can finish cleaning up later.
- Get the slicing chamber from the freezer. Make sure the magnetised block is dry and the glue hasn't hardened.
- Use the perforated spoon to carefully lift the brain out of the beaker. Take extra care not to push it against the glass wall. Gently place on the filter paper in the petri dish. Add enough slush of slicing ACSF to cover it. Use the brush to carefully remove any slush that remains stuck to the brain, so that it is free. Re-position the brain so that it sits on the cut you made in the frontal part of the brain (i.e. the cerebellum is facing up to you).
- Put glue on the magnetised block. Make sure there is enough to glue the brain, but not too much that the brain sinks on it.

Citation: Oriol Pavon Arocas, Tiago Branco Preparation of acute midbrain slices containing the superior colliculus and periaqueductal gray for patch-clamp recordings https://dx.doi.org/10.17504/protocols.io.8epv51pz5l1b/v6

This is an open access protocol distributed under the terms of the **Creative Commons Attribution License** (https://creativecommons.org/licenses/by/4.0/), which permits unrestricted use, distribution, and reproduction in any medium,

- With the spatula on your dominant hand and the brush on the other, carefully load the brain on the spatula so that the frontal side faces down (i.e. the bit where the olfactory bulb would have been is in contact with the spatula, and the cerebellum is facing up towards you). Next, carefully lift the brain and gently touch a clean tissue paper to dry out any excess liquid (this will ensure the tissue sticks to the glue). With the brush, gently transfer the brain from the spatula to the magnetised block, placing it on the glue. Use the brush to gently press the cortex down to ensure the brain properly sticks to the magnetised block (only do that if you don't care about the cortex). **N.B**: be very gentle when transferring the brain to the block. Do not hit the magnetised block against the table (e.g. to force the brain to get pressed against the glue) and avoid any unnecessary mechanical stress to the tissue.
- Rapidly use the glass pipette to pour some ice-cold slicing ACSF from the petri dish over the brain - this will both keep the brain cold and harden the glue.
- Carefully mount the magnetised block on the slicing chamber and proceed to fill the chamber with ice-cold slicing ACSF. Use the perforated spoon to protect the brain, so that the slush doesn't fall on it too hard (minimise mechanical stress).
- Place the slicing chamber on the vibratome and lock it.

Slicing 40m

5

This section details the slicing procedure using two different machines: a Leica VT1200S and a Campden Ci7000smz-2. Both provide good quality slices. Note that the steps detailed below will need to be adjusted if a different model or instrument is used.

5.1 **[OPTION 1 - Slicing with a Leica VT1200S]**

- Mount the slicing chamber to the vibratome. Fix a small bubbler to the slicing chamber and immerse it in the slicing ACSF to constantly equilibrate the solution with carbogen during slicing.
- With the brush, move the slush away from the brain.
- Lower the blade to horizontal position.
- Bring the slicing chamber up by pressing "UP" until the top of the brain is at the level of the blade.
- Press the button to move the blade closer to the brain.
- Set the setting to "Manual" and see below for a note on how to slice in this mode.

In the "Manual" mode of the Leica VT1200S, slice as follows: (1) press "RUN/STOP" to start slicing, (2) set the desired speed with the top wheel, (3) press "RUN/STOP" to stop slicing, (4) use the bottom wheel to bring the

Citation: Oriol Pavon Arocas, Tiago Branco Preparation of acute midbrain slices containing the superior colliculus and periaqueductal gray for patch-clamp recordings https://dx.doi.org/10.17504/protocols.io.8epv51pz5l1b/v6

This is an open access protocol distributed under the terms of the **Creative Commons Attribution License** (https://creativecommons.org/licenses/by/4.0/), which permits unrestricted use, distribution, and reproduction in any medium,

slicing chamber "DOWN" for 100 microns (i.e. away from the blade, so that you are no longer touching/pushing the brain down and you don't compress the tissue when collecting the slice and retracting the blade), (5) press the button to move the blade back (i.e. away from the brain) until completely out, (6) use the bottom wheel to bring the slicing chamber "UP" for 350 microns (i.e. set the desired slice thickness remembering you first moved 100 microns in the opposite direction, so the thickness of this slice will be 250 microns), (7) press "RUN/STOP" to resume slicing. Repeat until you collected all the slices you need.

- At the beginning, set the slicing speed to 0.10 mm/s and the amplitude to 1 mm. In the interest of time, slice thicker sections (500 to 900 microns) at this speed until you reach the PAG (or the area of interest).
- Once you start to see the Aqueduct, you are getting close to the PAG. Set the slice thickness to 250 microns and speed to 0.05 mm/s (see note above for details, keep the amplitude at 1 mm).
- While slicing, and as long as you don't need to keep it for your experiment, you can use the brush to carefully get rid of the cortex.
- Once the blade has surpassed the PAG, stop the slicing and bring the slicing chamber 100 microns down (see note above for details).
- Use the needle to carefully cut the slice at the bottom to separate it from the brain, making sure you don't touch the sharp end of the blade and without excessively pressing on the blade. Cut away a small piece of tissue from the bottom right side of the slice, so you can orient the slice in the scope and identify the right and left hemispheres.
- Using the glass pipette, collect the slice and transfer to the first recovery chamber. According to Bischofberger, et al., 2006 it is better to place the slice upside down from how it was sliced, as the tissue is healthier there.
- Proceed to obtain the next slice. Repeat the cycle until done. Preparing 250 microns thick slices should give you at least 4-5 usable slices containing the PAG.
- Try to place the slices on the recovery chamber following the order of slicing. This will allow you to easily locate the coordinates of each slice and keep track of which are the freshest slices.
- Given that the slicing speed is very low, you can start cleaning the tools and glassware you have finished using. Rinse everything with ddH_2O and dry. Put items and tools back to where they belong. Leave the glassware in the drying rack until baking.
- Once all the slices have been collected and are being incubated, proceed to clean the vibratome. Carefully remove the blade and thoroughly rinse the blade holder with ddH_2O to remove any residues of slicing ACSF (make sure the slicing chamber is still underneath so it collects the water). Remove the slicing chamber and take the magnetised block with the rest of the brain out - use the blade to scrape off the glue and the leftover tissue. Dispose of the blade in a sharps bin. Disassemble the slicing chamber and thoroughly wash with ddH_2O. Leave to dry.
- Using a piece of paper, carefully dry the blade holder and the vibratome. Place

Citation: Oriol Pavon Arocas, Tiago Branco Preparation of acute midbrain slices containing the superior colliculus and periaqueductal gray for patch-clamp recordings https://dx.doi.org/10.17504/protocols.io.8epv51pz5l1b/v6

This is an open access protocol distributed under the terms of the **Creative Commons Attribution License** (https://creativecommons.org/licenses/by/4.0/), which permits unrestricted use, distribution, and reproduction in any medium,

a piece of filter paper where the blade slots in, so it absorbes any water left. Lower the blade holder and switch the vibratome off.

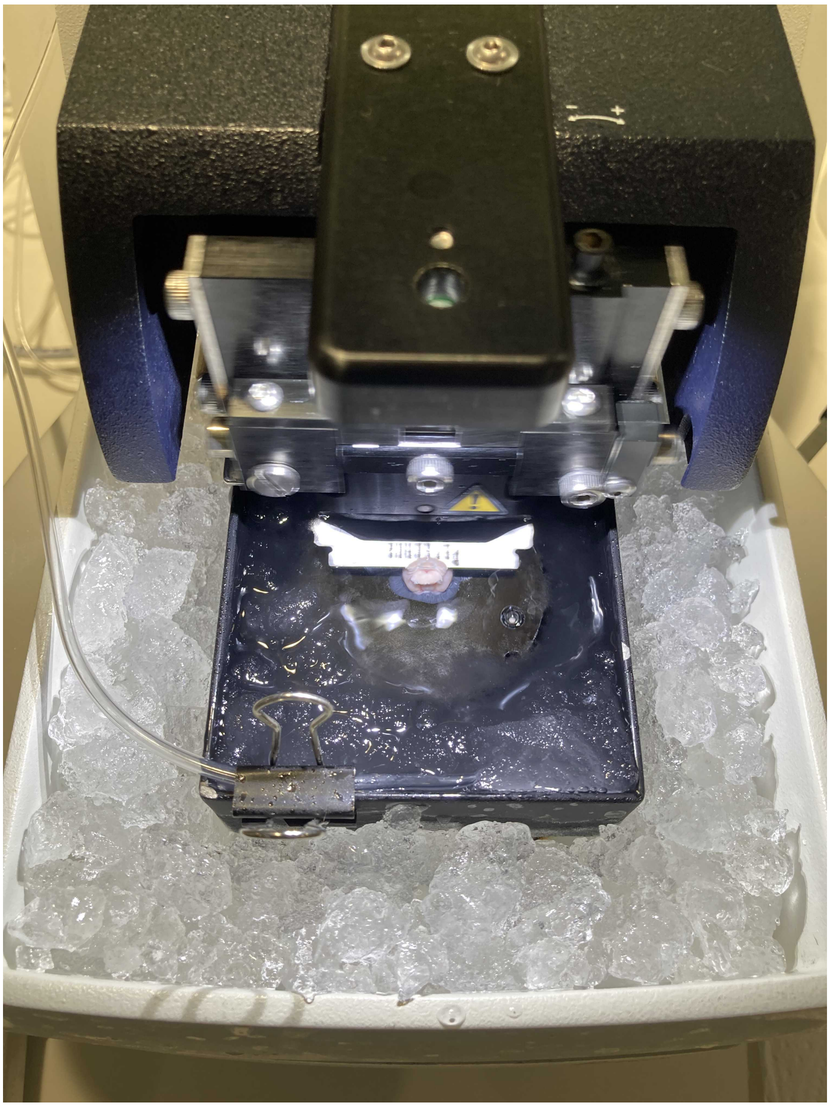

Figure S12. Preparation of coronal acute brain slices with a Leica VT1200S.

5.2 [OPTION 2 - Slicing with a Campden Ci7000smz-2]

- Switch vibratome ON (at the back). The advantage of using this vibratome is that you do not need to calibrate or align the blade each time, as it uses a ceramic blade that only needs calibrating when you first mount it or replace with a new one. Note that it is also possible to use ceramic blades on the Leica.
- Mount the slicing chamber, being careful not to touch the blade.
- Fix a small bubbler to the slicing chamber and immerse it in the slicing ACSF to constantly equilibrate the solution with carbogen during slicing.
- Select the slicing program (N.B. you need to have created it in advance).
- With the brush, move the slush away from the brain.

Citation: Oriol Pavon Arocas, Tiago Branco Preparation of acute midbrain slices containing the superior colliculus and periaqueductal gray for patch-clamp recordings https://dx.doi.org/10.17504/protocols.io.8epv51pz5l1b/v6

This is an open access protocol distributed under the terms of the **Creative Commons Attribution License** (https://creativecommons.org/licenses/by/4.0/), which permits unrestricted use, distribution, and reproduction in any medium,

- Press "Load Bath" for a few seconds - the chamber will go up.
- Press "Height UP". Then press "Advance", followed by "Slice ON" - this will approach the blade to the brain. Stop when close but before touching the brain. This point will be recorded and the blade will go back there each time you press "Return" during the slicing process.
- The thickness of the slice is predetermined in the program, and in our case it is set to 250 microns.
- To obtain each slice, follow the sequence of pressing "Slice ON - Slice OFF - Return". Start slicing at a speed of 0.10 mm/s and repeat the sequence as many times as needed until you reach the PAG or area of interest (each cycle goes down a predetermined value, in our case 250 microns).
- Once in the area of interest, press "Slice ON" and adjust speed to 0.04-0.06 mm/s. While slicing, and as long as you don't need to keep it for your experiment, you can use the brush to carefully get rid of the cortex.
- Once the blade has surpassed the PAG, press "Slice OFF". Use the needle to carefully cut the slice at the bottom to separate it from the brain, making sure you don't touch the sharp end of the blade and without excessively pressing on the blade. Cut away a small piece of tissue from the bottom right side of the slice, so you can orient the slice in the scope and identify the right and left hemispheres.
- Using the glass pipette, collect the slice and transfer to the first recovery chamber. According to Bischofberger, et al., 2006 it is better to place the slice upside down from how it was sliced, as the tissue is healthier there.
- Try to place the slices on the recovery chamber following the order of slicing. This will allow you to easily locate the coordinates of each slice and keep track of which are the freshest slices.
- Press "Return": this takes the blade back to the starting point, and lowers it 250 microns for the next slice.
- Proceed to obtain the next slice. Repeat the cycle until done. Preparing 250 microns thick slices should give you at least 4-5 usable slices containing PAG.
- Given that the slicing speed is very low, you can start cleaning the tools and glassware you have finished using. Rinse everything with ddH2O and dry. Put items and tools back to where they belong. Leave the glassware in the drying rack until baking.
- Once all the slices have been collected and are being incubated, proceed to clean the vibratome. Press "Load Bath" for a few seconds - the chamber will go down.
- Thoroughly rinse the blade with ddH2O to remove any residues of slicing ACSF (make sure the slicing chamber is still underneath so it collects the water).
- Remove the slicing chamber and take the magnetised block with the rest of the brain out - use a blade to scrape off the glue and the leftover tissue.
- Thoroughly clean the slicing chamber with ddH2O. Leave to dry.
- Using a piece of paper, carefully dry the blade from the back (never touch the sharp edge).
- Place safety cover on the blade (a red plastic piece with a magnet).
- Switch vibratome OFF.

[Common considerations - Clean up]

Citation: Oriol Pavon Arocas, Tiago Branco Preparation of acute midbrain slices containing the superior colliculus and periaqueductal gray for patch-clamp recordings https://dx.doi.org/10.17504/protocols.io.8epv51pz5l1b/v6

This is an open access protocol distributed under the terms of the **Creative Commons Attribution License** (https://creativecommons.org/licenses/by/4.0/), which permits unrestricted use, distribution, and reproduction in any medium,

5.3

- Rinse the bubblestones you have used with ddH2O and immerse in a beaker with ddH2O. Leave them bubbling in ddH2O until the end of the incubation to get rid of any salt residues.
- Make sure to properly clean all the tools, glassware, and instruments you used. Carefully dry and put them back to where they belong.
- Squirt some ddH2O on the bench where you have done the brain extraction, around the vibratome, and anywhere slicing ACSF may have fallen. Wipe surfaces and dry.

Incubation 1h

6

This section covers the different incubation strategies followed for each set of solutions described in Section 2 and used in either Path A or B. It may be worth trying slightly different durations and temperatures to find the appropriate conditions for your particular brain area and animal age. It may also be a good idea to monitor how the room temperature changes throughout the year, as this could have an impact on the incubation steps done at room temperature.

6.1 **[PATH A - Decapitation under terminal isoflurane anaesthesia]**

In this path, the first recovery chamber contains slicing ACSF (sucrose) and the second recovery chamber contains recording ACSF. Both have been heated to 35°C and equilibrated with carbogen until now. Any bubbles stuck to the net have been removed prior to transferring the slices. Once all the slices have been transferred to the first recovery chamber, proceed with the incubation as follows:

- Incubate slices for 30 min in slicing ACSF (sucrose) at 35°C, constantly equilibrated with 95% O2 and 5% CO2.
- After 30 min, carefully remove the second recovery chamber containing recording ACSF from the heating block and place on the bench at room temperature. Use the glass pipette to transfer each slice from the first to the second recovery chamber (from slicing to recording ACSF). Try to minimise any solution carryover when transferring the slices (you want as little sucrose and magnesium as possible added to the new solution).
- Once all the slices have been transferred to the second recovery chamber, let equilibrate to room temperature for at least 30 more minutes before recording. Constantly equilibrate the solution with 95% O2 and 5% CO2.
- If the slices express fluorescence or channelrhodopsin, cover with aluminum foil to protect from ambient light.

6.2 **[PATH B - Perfusion with ACSF under terminal isoflurane anaesthesia]**

Citation: Oriol Pavon Arocas, Tiago Branco Preparation of acute midbrain slices containing the superior colliculus and periaqueductal gray for patch-clamp recordings https://dx.doi.org/10.17504/protocols.io.8epv51pz5l1b/v6

This is an open access protocol distributed under the terms of the **Creative Commons Attribution License** (https://creativecommons.org/licenses/by/4.0/), which permits unrestricted use, distribution, and reproduction in any medium,

In this path, the first recovery chamber contains slicing ACSF (NMDG-HEPES) heated to 32°C and constantly equilibrated with carbogen. The second recovery chamber contains holding ACSF (HEPES) and has been heated to 32°C until right before commencing the slicing procedure and then removed from the heating block and placed on the bench at room temperature, constantly equilibrated with 95% O2 and 5% CO2.

In this case, the incubation procedure is slightly different, and the incubation duration is timed independently for each slice.

- Incubate each slice for 10-12 min in slicing ACSF (NMDG-HEPES) at 32°C, constantly equilibrated with 95% O2 and 5% CO2.
- After the 10-12 min, carefully transfer each slice to the second recovery chamber containing holding ACSF (HEPES), trying to minimise solution carryover. Incubate for at least 1 h at room temperature before recording, constantly equilibrating the solution with 95% O2 and 5% CO2.
- If the slices express fluorescence or channelrhodopsin, cover with aluminum foil to protect from ambient light.

6.3 **[Common considerations]**

Once the incubation period is over, use the glass pipette to collect the slice you will start recording from and transfer it to the recording chamber in your rig. To stably hold the slice in place you can place a platinum ring with equidistant nylon threads on top.

- It is important to make sure any holding/slicing solution is washed out before you start your recordings. The slice should be perfused for a sufficiently long time (at least 10 min for a flow rate of 2–5 mL/min) with recording ACSF before starting your experiment. This is important because the different compositions between the recording and the holding/slicing ACSF can difficult obtaining good seals and can affect both excitability and synaptic transmission.

Clean up 15m

7

Regardless of whether Path A or B was followed, the last step before recording is cleaning up. Most points have already been mentioned in the corresponding sections, but they are collected and listed together below.

7.1
- **Tools**: thoroughly wash all tools and glassware with ddH2O, making sure to get rid of any blood and salts residues. If any of the tools used during brain extraction have been in contact with glue, use a blade to scrape it off before

Citation: Oriol Pavon Arocas, Tiago Branco Preparation of acute midbrain slices containing the superior colliculus and periaqueductal gray for patch-clamp recordings https://dx.doi.org/10.17504/protocols.io.8epv51pz5l1b/v6

This is an open access protocol distributed under the terms of the **Creative Commons Attribution License** (https://creativecommons.org/licenses/by/4.0/), which permits unrestricted use, distribution, and reproduction in any medium,

cleaning and drying the tool.

- **Consumables:** properly dispose of any consumables or PPE that have been contaminated by blood or organic tissue using the appropriate bin.
- Properly dispose of any **sharps**: needles, blades, and broken glass go to a sharps bin.
- Properly dispose of the carcass according to the guidelines and protocols in place at the home institution.
- **Clean the bubblestones:** rinse with ddH2O and then transfer to a beaker with fresh ddH2O. Immerse them and leave them bubbling for a while. At the end of the day, transfer to a separate beaker with a piece of clean tissue paper at the bottom and leave to dry. Switch carbogen off.
- **Glassware:** thoroughly wash all glassware with ddH2O to remove any salt residues from the ACSF solutions. Leave in the rack to dry until the end of the experiment. If possible, proceed to sterilise by baking at 200°C for 2 h, to make sure bacteria can't grow there. If the oven has a timer, you can do this at the end of the day so that it has time to cool down and all glassware is sterile and ready to be used the next morning.
- **Vibratome:** carefully remove the blade and thoroughly rinse the blade holder with ddH2O to remove any salts (make sure the slicing chamber is still underneath so it collects the water). Remove the slicing chamber and take the magnetised block with the rest of the brain out - use the blade to scrape off the glue and the rest of the tissue. Dispose of the blade in a sharps bin. Disassemble the slicing chamber and thoroughly wash with ddH2O. Leave to dry. Using a piece of paper, carefully dry the blade holder and the vibratome. Place a piece of filter paper where the blade slots in, so it dries up any water left. Lower the blade holder and switch vibratome off.
- **Bench surfaces:** spray some ddH2O on the bench where you have done the brain extraction, the area around the vibratome, and anywhere slicing ACSF may have fallen. Wipe surfaces and dry.

Troubleshooting slice quality problems

8

This section highlights some of the key aspects to check when troubleshooting slice quality problems. One important thing to keep in mind when troubleshooting is to only change one thing at a time. In addition, it may be a good idea to invest time testing different combinations of blade, brain extraction method (perfusion or decapitation), solutions, and incubation times to find what works best for your brain area, animal age, and experimental conditions.

8.1 [Slicing]

An important question when troubleshooting slice quality is: do the slices look good immediately after slicing? What about after the incubation is over? Do they look good immediately after you place them in the recording chamber, or do they

Citation: Oriol Pavon Arocas, Tiago Branco Preparation of acute midbrain slices containing the superior colliculus and periaqueductal gray for patch-clamp recordings https://dx.doi.org/10.17504/protocols.io.8epv51pz5l1b/v6

This is an open access protocol distributed under the terms of the **Creative Commons Attribution License** (https://creativecommons.org/licenses/by/4.0/), which permits unrestricted use, distribution, and reproduction in any medium,

already look unhealthy then? The answers to these questions will point you to which of the steps is more likely to be the problem.

- If the slices look already unhealthy right after slicing (i.e. with a high proportion of very contrasty cells and "fried eggs"), you can start by checking the slicing solutions (double check the pH and osmolality or try making a new batch), the vibratome (perhaps it needs servicing or the blade was not properly aligned), or your brain extraction technique (you may need to do it faster or be more careful, or you may need to try intracardially perfusing the animal with slicing ACSF).
- If the slices appear healthy under the microscope right after slicing, but look unhealthy after incubation, you probably have an issue with either the incubation time, temperature, or solution. It could also be that the recovery chamber is contaminated or the solution is not sufficiently oxygenated.
- Finally, if the slices look perfect right after the incubation, but degrade quickly once you put them in the recording chamber and start your experiment, you may be having issues with the recording ACSF, the temperature in the bath (malfunctioning heater), the oxygenation of the solution, or excitotoxicity of the tissue (recording at room temperature should reduce this).

Other guidelines and advice that may be useful when trying to improve the slicing procedure:

- When troubleshooting, try to use younger animals first (<6 weeks old if possible). Tissue from young animals is usually more forgiving to the procedure and will help you find what the major problem is.
- When extracting the brain, keep the temperature low (0-4°C) and dissect as fast and as careful as you can. Make sure to immerse the head and brain in ice-cold solution at each step to keep as cold as possible.
- If you are not already doing it, try to intracardially perfuse the animals with ice-cold slicing ACSF before extracting the brain. This is the best way to clear the blood out of the brain by replacing it with the solution of your choice (e.g. low sodium and calcium and high magnesium and NMDG) to rapidly slow metabolic activity and reduce excitotoxicity. Again, this may work better or worse depending on the brain area and age of the animals you work on, so it is worth comparing the results from intracardial perfusion to those from decapitation before deciding on one approach.
- The exact duration of the recovery period is critical for obtaining the optimal tissue preservation. Try to compare the results obtained with different incubation times to find what works best for your particular brain area and experimental conditions.
- Regardless of whether you perform your recordings at room or physiological temperature, the incubation step at room temperature is crucial as it allows the slices to recover from spine sprouting, which occurs when slicing (see Edwards, et al., 1989).
- If possible, check the slice quality of a colleague using the same equipment and solutions than you. This can help you rule out problems with carbogen,

Citation: Oriol Pavon Arocas, Tiago Branco Preparation of acute midbrain slices containing the superior colliculus and periaqueductal gray for patch-clamp recordings https://dx.doi.org/10.17504/protocols.io.8epv51pz5l1b/v6

This is an open access protocol distributed under the terms of the **Creative Commons Attribution License** (https://creativecommons.org/licenses/by/4.0/), which permits unrestricted use, distribution, and reproduction in any medium,

water, or slicing machine. However, beware of differences between brain areas (the same procedure can give an excellent quality for visual cortex and kill all your cells in the periaqueductal gray).

8.2 **[Vibratome]**

It is very important that the machine you use to prepare slices is of a high quality and in excellent condition.

- If possible, try to have the vibratome you use routinely serviced.
- Always calibrate the z displacement of the blade to make sure it is not inflicting unwanted mechanical damage on your slices.
- Try using ceramic blades, but beware they may or may not improve your slice quality depending on the brain region you study. If you opt to use them, replace the blade if old or blunted.
- Change the angle or the speed at which you slice - again, the usefulness of this may depend on the brain region you work on. The structure of the periaqueductal gray makes the coronal slice the best option, but this may not be the case for neighbouring areas.
- If you are not sure your vibratome is the problem, you could try to find a colleague in a neighbouring laboratory who currently gets good slices and ask whether you may use their machine to rule out there is an issue with yours.

8.3 **[Solutions]**

- If working with animals over 8 weeks old, try to use solutions optimised for adult brain tissue such as NMDG- or Choline-based ACSF, where NMDG-Cl or Choline-Cl replaces NaCl. Together with a low temperature, this will help reduce excitotoxicity. It may be worth comparing the results between the high sucrose slicing solution and the NMDG/Choline solution in your hands.
- Adding 20 mM HEPES can effectively provide stronger pH buffering in the physiological range and prevent excessive edema.
- You can also try adding supplements for energy supply and antioxidant function. Myo-inositol and sodium pyruvate provide additional entry points into cellular metabolism, whereas ascorbic acid, thiourea, and N-acetyl-L-cysteine (a cell-permeable glutathione precursor) help reduce oxidative stress.
- Use purified water that is free of contaminants such as trace metals. Impurities like these are often found in distilled water sources and cause damage to the slices and can even convert anti-oxidants such as ascorbate into pro-oxidants.
- Make sure your solutions are fresh, ideally less than 24 h but certainly not older than a week.
- Minimise carryover when transferring slices between solutions: an extreme way of doing this would be having an intermediate beaker (so you transfer the slice to the first beaker, and then from there to the second) so that any carryover is diluted.
- If you suspect your solutions are the cause of low quality slices, you could try to find a colleague in a neighbouring laboratory who currently gets good slices

Citation: Oriol Pavon Arocas, Tiago Branco Preparation of acute midbrain slices containing the superior colliculus and periaqueductal gray for patch-clamp recordings https://dx.doi.org/10.17504/protocols.io.8epv51pz5l1b/v6

This is an open access protocol distributed under the terms of the **Creative Commons Attribution License** (https://creativecommons.org/licenses/by/4.0/), which permits unrestricted use, distribution, and reproduction in any medium,

and ask whether you could use some of their solutions for a test. Alternatively, you could ask whether you could use a spare slice from them to check they are equally happy in your rig and theirs.

8.4 [Water]

Another usual suspect when troubleshooting slice quality issues is the water you use to prepare solutions. It is possible that the water source has become contaminated or the filters saturated.

- If in doubt, and if possible, you can purchase commercially available ultrapure ddH2O and make fresh solutions with it. One of the first things to rule out should always be the batch of ACSF you used.
- Make sure all the solutions and concentrated stocks are made with ultrapure, double-distilled water.
- It is advisable to regularly submit samples from your water system for analysis to check for bacterial contamination and to compare the quality to UltraPure (e.g. Severn Biotech) and Molecular Grade Sterile Water (e.g. Corning).
- If using a water purification system, ensure it is properly maintained: replace filters, run a cleaning and sterilization cycle, replace UV lamps, etc.

8.5 [Salts]

Although infrequent, it is possible that the salts and chemicals you use to prepare the solutions have gone bad or you received a contaminated batch.

- Some salts (such as sodium pyruvate) can go bad quicker than others. Keep track of the opening date and replace if older than 3-6 months.
- If nothing else works, you can try to replace all salts for new ones and make fresh solutions.

8.6 [Glassware]

A very important point is to avoid bacterial contamination and salt build up in your glassware.

- To minimise this risk, thoroughly clean the glassware and tools you use to prepare solutions, with special emphasis on the recovery chambers.
- If possible, have a separate set of glassware specifically dedicated to your slice work.
- If you dispose of an oven, sterilise the glassware by baking it for 2 h at 200°C before every use.
- It is also possible to use a solution with 0.1 M nitric acid to help get rid of any salt build up on the glass, but make sure you thoroughly clean with ultrapure ddH2O afterwards.

8.7 [Carbogen]

Citation: Oriol Pavon Arocas, Tiago Branco Preparation of acute midbrain slices containing the superior colliculus and periaqueductal gray for patch-clamp recordings https://dx.doi.org/10.17504/protocols.io.8epv51pz5l1b/v6

This is an open access protocol distributed under the terms of the **Creative Commons Attribution License** (https://creativecommons.org/licenses/by/4.0/), which permits unrestricted use, distribution, and reproduction in any medium,

It is also possible that the solutions and slices are not sufficiently oxygenated.

- Set the bubbling really high whenever possible to ensure you oxygenate the tissue as much as possible, and always equilibrate solutions for 20-30 min prior to use.
- If not sure, test the carbogen outlets to ensure the mixture has the right concentration (95% O2 and 5% CO2).
- If using tanks, make sure that the current one is not empty or that you did not get a bad batch.

8.8 **[Osmolality, pH, and temperature]**

- Make sure the osmometer and pH meter are properly calibrated and clean.
- Always check the osmolality and pH of your solutions, as both values may change after 30 minutes of bubbling. When possible, cover the beakers and recovery chambers with parafilm to minimise evaporation and loss of solution when bubbling.
- If you need to reduce osmolality of the slicing solution, try to change the amount of sucrose or NMDG/Choline, so that you don't modify the other ion concentration.
- pH is temperature dependent and slicing ACSF can turn acidic after bubbling for 30 min on ice (0-4°C). Do check if that is the case and readjust if necessary so it is as close to 7.3-7.4 as possible. A low pH will certainly have an effect on the quality of the slices.
- If possible, use an external thermometer to double-check the temperature in the bath, recovery chamber, and recording chamber is what you are expecting.
- Surround the slicing chamber with ice and keep it in the freezer until use to keep the temperature as low as possible.

8.9 **[PFA contamination]**

- Do your best to avoid and rule out an accidental PFA contamination in your tubing, bench area, glassware, tools. Make sure you work with tools and on a laboratory area dedicated to slice work whenever possible, including for perfusing the animals and extracting the brain.

8.10 **[Example of a slice troubleshooting cycle]**

Sometimes, you may be at a loss as to what is affecting the quality of your slices. In such occasions, a systematic approach or knowing what worked for others may help identify the issue. As an example, we tried the following changes to solve our slice quality issues after a series of bad experiments:

- First, we switched to using commercially available bottled ultrapure water to make solutions (a previous troubleshooting cycle ended when discovering a problem with the water source we were using), and proceeded to clean and

Citation: Oriol Pavon Arocas, Tiago Branco Preparation of acute midbrain slices containing the superior colliculus and periaqueductal gray for patch-clamp recordings https://dx.doi.org/10.17504/protocols.io.8epv51pz5l1b/v6

This is an open access protocol distributed under the terms of the **Creative Commons Attribution License** (https://creativecommons.org/licenses/by/4.0/), which permits unrestricted use, distribution, and reproduction in any medium,

calibrate the pH meter and osmometer.

- We ordered new glassware and thoroughly washed it, and we started baking it to sterilise it.
- We replaced all bubblestones and tubing, renewed all salts, made new stocks (e.g. 2.5 M KCl and 1.25 M NaH_2PO_4), made new recovery and recording chambers, and flushed the tubing of the perfusion system in the rig with EtOH and ddH_2O.
- We tested the water source and carbogen outlets, and serviced the vibratome. We asked to use a new vibratome from a neighbouring laboratory until ours could be serviced.
- We started intracardially perfusing our animals with ACSF, as many studies have shown this helps preserve the tissue in better condition (especially in adult animals over 6-8 weeks old), and tried more protective solutions (NMDG and Choline based). Effectively, we moved from Path A to Path B of this protocol.
- We watched how several colleagues sliced and compared their approach and solutions to ours. We introduced further recommendations such as testing and adjusting the pH right before use (ACSF solutions turned acidic when cold).
- We compared different approaches by slicing pairs of younger animals (~4 weeks old) and changing one thing at a time: one perfused vs. one decapitated, one with a longer incubation vs. one with a shorter, one sliced with a vibratome vs. one sliced with a different machine, one with our usual solution vs. one with a new recipe, one with the usual blade vs. one with a new ceramic blade, etc.
- After a series of rounds of testing, and after always sticking with the best option of each comparison, we found we obtained the best improvements after servicing the vibratome (it turned out it had a malfunctioning piece), changing the solutions (NMDG and HEPES holding gave significantly better results than sucrose), and intracardially perfusing the animals.

8.11 [Literature and resources used]

- Edwards FA, Konnerth A, Sakmann B, Takahashi T. A thin slice preparation for patch clamp recordings from neurones of the mammalian central nervous system. Pflügers Arch Eur J Physiol. 1989;414(5):600–12. DOI: https://doi.org/10.1007/BF00580998
- Gibb AJ, Edwards FA. Patch clamp recording from cells in sliced tissues. In: Ogden DC, editor. Microelectrode techniques, The Plymouth workshop handbook. 2nd ed. Cambridge: The Company of Biologists Limited; 1994. p. 255–74
- Lipton P, Aitken PG, Dudek FE, Eskessen K, Espanol MT, Ferchmin PA, et al. Making the best of brain slices: comparing preparative methods. J Neurosci Methods. 1995;59(1):151–6. DOI: https://doi.org/10.1016/0165-0270(94)00205-U
- Aitken PG, Breese GR, Dudek FF, Edwards F, Espanol MT, Larkman PM, et al. Preparative methods for brain slices: a discussion. J Neurosci Methods. 1995;59(1):139–49. DOI: https://doi.org/10.1016/0165-0270(94)00204-T
- Moyer JR, Brown TH. Methods for whole-cell recording from visually

Citation: Oriol Pavon Arocas, Tiago Branco Preparation of acute midbrain slices containing the superior colliculus and periaqueductal gray for patch-clamp recordings https://dx.doi.org/10.17504/protocols.io.8epv51pz5l1b/v6

This is an open access protocol distributed under the terms of the **Creative Commons Attribution License** (https://creativecommons.org/licenses/by/4.0/), which permits unrestricted use, distribution, and reproduction in any medium,

preselected neurons of perirhinal cortex in brain slices from young and aging rats. J Neurosci Methods. 1998;86(1):35–54. DOI: https://doi.org/10.1016/S0165-0270(98)00143-5

- Brahma B, Forman RE, Stewart EE, Nicholson C, Rice ME. Ascorbate inhibits edema in brain slices. J Neurochem. 2000;74(3):1263–70. DOI: https://doi.org/10.1046/j.1471-4159.2000.741263.x
- MacGregor DG, Chesler M, Rice ME. HEPES prevents edema in rat brain slices. Neurosci Lett. 2001;303(3):141–4. DOI: https://doi.org/10.1016/S0304-3940(01)01690-1
- Bischofberger J, Engel D, Li L, Geiger JRP, Jonas P. Patch-clamp recording from mossy fiber terminals in hippocampal slices. Nat Protoc. 2006;1(4):2075–81. DOI: https://doi.org/10.1038/nprot.2006.312
- Tanaka Y, Tanaka Y, Furuta T, Yanagawa Y, Kaneko T. The effects of cutting solutions on the viability of GABAergic interneurons in cerebral cortical slices of adult mice. J Neurosci Methods. 2008;171(1):118–25. DOI: https://doi.org/10.1016/j.jneumeth.2008.02.021
- Huang S, Uusisaari MY. Physiological temperature during brain slicing enhances the quality of acute slice preparations. Front Cell Neurosci. 2013;7(APR):1–8. DOI: https://doi.org/10.3389/fncel.2013.00048
- Ting JT, Daigle TL, Chen Q, Feng G. Acute brain slice methods for adult and aging animals: Application of targeted patch clamp analysis and optogenetics. In: Martina M, Taverna S, editors. Methods in Molecular Biology. New York, NY: Springer New York; 2014. p. 221–42. (Methods in Molecular Biology; vol. 1183). DOI: https://doi.org/10.1007/978-1-4939-1096-0_14
- Buskila Y, Breen PP, Tapson J, van Schaik A, Barton M, Morley JW. Extending the viability of acute brain slices. Sci Rep. 2015;4(1):5309. DOI: https://doi.org/10.1038/srep05309
- Ting JT, Kalmbach B, Chong P, De Frates R, Keene CDi, Gwinn RP, et al. A robust ex vivo experimental platform for molecular-genetic dissection of adult human neocortical cell types and circuits. Sci Rep. 2018;8(1):8407. DOI: http://dx.doi.org/10.1038/s41598-018-26803-9
- Ting JT, Lee BR, Chong P, Soler-Llavina G, Cobbs C, Koch C, et al. Preparation of Acute Brain Slices Using an Optimized N-Methyl-D-glucamine Protective Recovery Method. J Vis Exp. 2018;(132):1–13. DOI: https://dx.doi.org/10.3791/53825
- Gouwens NW, Sorensen SA, Berg J, Lee C, Jarsky T, Ting J, et al. Classification of electrophysiological and morphological neuron types in the mouse visual cortex. Nat Neurosci. 2019;22(7):1182–95. DOI: https://doi.org/10.1038/s41593-019-0417-0
- Mandelbaum G, Taranda J, Haynes TM, Hochbaum DR, Huang KW, Hyun M, et al. Distinct Cortical-Thalamic-Striatal Circuits through the Parafascicular Nucleus. Neuron. 2019;102(3):636-652.e7. DOI: https://doi.org/10.1016/j.neuron.2019.02.035
- Dorst MC, Tokarska A, Zhou M, Lee K, Stagkourakis S, Broberger C, et al. Polysynaptic inhibition between striatal cholinergic interneurons shapes their network activity patterns in a dopamine-dependent manner. Nat Commun. 2020;11(1):5113. DOI: http://dx.doi.org/10.1038/s41467-020-18882-y
- Eguchi K, Velicky P, Hollergschwandtner E, Itakura M, Fukazawa Y, Danzl JG,

Citation: Oriol Pavon Arocas, Tiago Branco Preparation of acute midbrain slices containing the superior colliculus and periaqueductal gray for patch-clamp recordings https://dx.doi.org/10.17504/protocols.io.8epv51pz5l1b/v6

This is an open access protocol distributed under the terms of the **Creative Commons Attribution License** (https://creativecommons.org/licenses/by/4.0/), which permits unrestricted use, distribution, and reproduction in any medium,

et al. Advantages of Acute Brain Slices Prepared at Physiological Temperature in the Characterization of Synaptic Functions. Front Cell Neurosci. 2020;14(March):35–7. DOI: https://doi.org/10.3389/fncel.2020.00063

- Gouwens NW, Sorensen SA, Baftizadeh F, Budzillo A, Lee BR, Jarsky T, et al. Integrated Morphoelectric and Transcriptomic Classification of Cortical GABAergic Cells. Cell. 2020;183(4):935-953.e19. DOI: https://doi.org/10.1016/j.cell.2020.09.057
- McCauley JP, Petroccione MA, D'Brant LY, Todd GC, Affinnih N, Wisnoski JJ, et al. Circadian Modulation of Neurons and Astrocytes Controls Synaptic Plasticity in Hippocampal Area CA1. Cell Rep. 2020;33(2):108255. DOI: https://doi.org/10.1016/j.celrep.2020.108255
- Bakken TE, Jorstad NL, Hu Q, Lake BB, Tian W, Kalmbach BE, et al. Comparative cellular analysis of motor cortex in human, marmoset and mouse. Nature. 2021;598(7879):111–9. DOI: https://doi.org/10.1038/s41586-021-03465-8
- Campagnola L, Seeman SC, Chartrand T, Kim L, Hoggarth A, Gamlin C, et al. Local connectivity and synaptic dynamics in mouse and human neocortex. Science. 2022;375(6585). DOI: https://doi.org/10.1126/science.abj5861

Other resources:

- Allen Institute Technical White Papers [link].
- "Tips for performing adult animal brain slicing for patch clampers", NeuroWire blog by Jonathan Ting [link].
- "Tips for improving your electrophysiology experiments", NeuroWire blog by Nour Al-muhtasib [link].

Patch-Clamp rig maintenance

9

This final section provides a list of tips and tricks for a good rig maintenance collected over time from advice from colleagues and from our own experience. This is by no means an exhaustive or complete list, but rather an attempt to keep a log of strategies and solutions that have worked in the past.

9.1 [Optics]

- **Camera calibration:** you can use a microruler to calibrate the camera on your rig. Take a picture (Figure S13) and measure the distance in pixels between a section of the microruler (e.g. using Fiji or ImageJ). Use the resolution of the camera and the microruler to obtain the pixel to micron relationship in order to be able to add the correct scale bar to any pictures you take. Make sure you do this for all the objectives you will use in your experiments.

Citation: Oriol Pavon Arocas, Tiago Branco Preparation of acute midbrain slices containing the superior colliculus and periaqueductal gray for patch-clamp recordings https://dx.doi.org/10.17504/protocols.io.8epv51pz5l1b/v6

This is an open access protocol distributed under the terms of the **Creative Commons Attribution License** (https://creativecommons.org/licenses/by/4.0/), which permits unrestricted use, distribution, and reproduction in any medium,

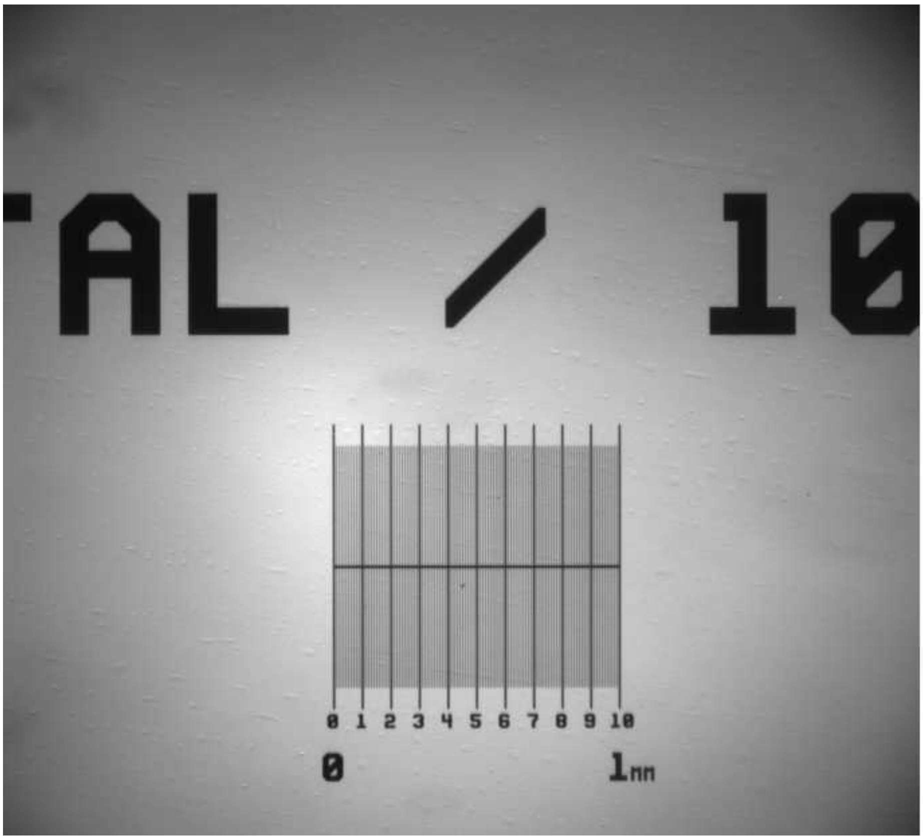

Figure S13. Example image with a microruler to calibrate the pixel size of your system.

- **Kohler illumination**: make sure you periodically focus and center the condenser to obtain Kohler illumination. Do this on the high magnification objective so that you have the optimum contrast and resolution when looking for cells to record from. There are plenty of guides on how to do this online, including these from MicroscopyU, Zeiss, Olympus, Scientifica, or Microscopist.
- There are also several online references to help you choose between oblique illumination, differential inference contrast illumination, and infrared light source (770 nm or 900 nm): [Oblique - MicroscopyU], [Oblique - Scientifica], [DIC - MicroscopyU], [DIC - Scientifica].

9.2 [Proper positioning of the inflow and outflow in the recording chamber]

Finding the optimal position for the inflow and outflow will ensure the bath level remains stable throughout your experiment.

- If using a needle as an outflow/suction, make sure the end is not completely immersed but it rather has half the tip immersed and the other half resting above the bath level (Figure S14). Obtaining the diameter and angle that best work in your set up may require several adjustments made by bending, cutting, and pressing the tip of the needle. The correct positioning should result in air bubbles constantly being formed in the outflow/suction, such that the bath level remains stable.

Citation: Oriol Pavon Arocas, Tiago Branco Preparation of acute midbrain slices containing the superior colliculus and periaqueductal gray for patch-clamp recordings https://dx.doi.org/10.17504/protocols.io.8epv51pz5l1b/v6

This is an open access protocol distributed under the terms of the **Creative Commons Attribution License** (https://creativecommons.org/licenses/by/4.0/), which permits unrestricted use, distribution, and reproduction in any medium,

- Once the positioning of the suction is good, you can modify the inflow/outlfow speed ratio (if using a pump that allows to independently change that) until you find the value at which the bath level minimally oscillates. If the bath level oscillates, you may end up with problems in the baseline of your recordings.
- Having the inflow closer to the bottom of the recording chamber (i.e. lower) and the outflow positioned a bit further up (i.e. higher) can also help to keep the bath level stable.
- Positioning the inflow and outflow opposite of each other will ensure the solution flows evenly through the chamber and the right concentration of ACSF (and any drug dissolved in it) reaches the slice.

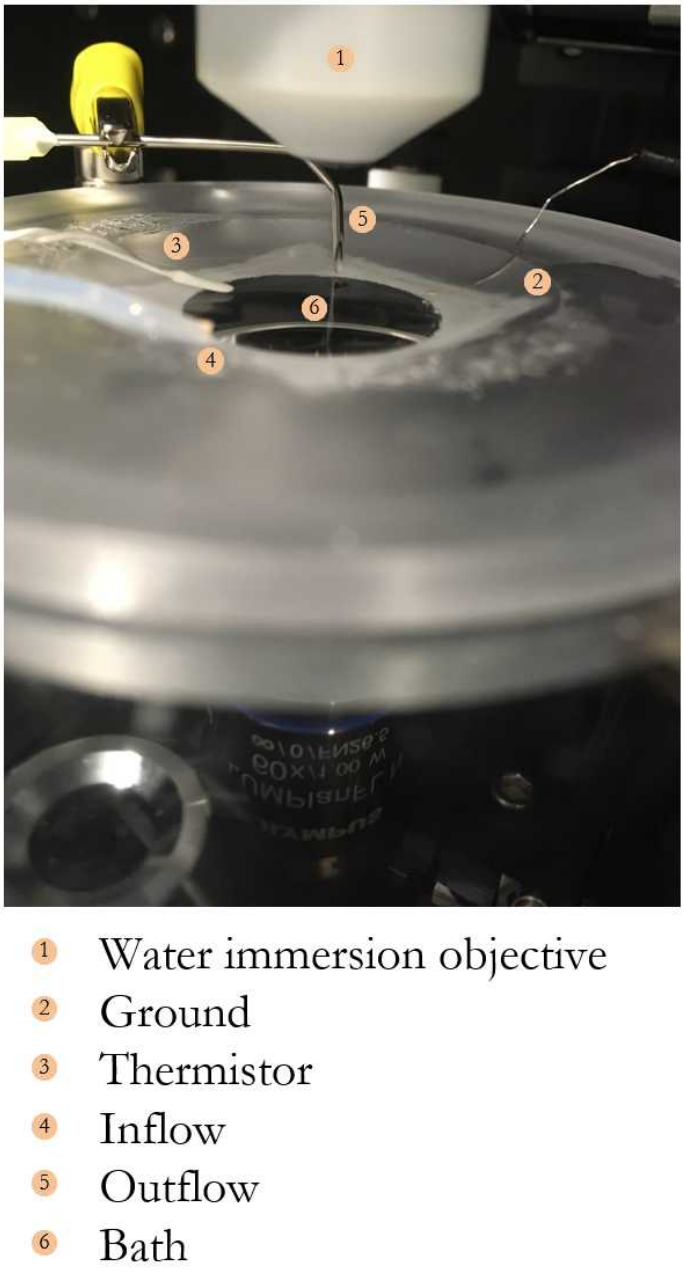

Figure S14. Overview of a recording chamber and its elements.

Citation: Oriol Pavon Arocas, Tiago Branco Preparation of acute midbrain slices containing the superior colliculus and periaqueductal gray for patch-clamp recordings https://dx.doi.org/10.17504/protocols.io.8epv51pz5l1b/v6

This is an open access protocol distributed under the terms of the **Creative Commons Attribution License** (https://creativecommons.org/licenses/by/4.0/), which permits unrestricted use, distribution, and reproduction in any medium,

Other considerations:

- To reduce pump-related noise, you can cut the tubing before it reaches the chamber, insert two needles to reconnect the tubing, and ground them.
- If using a pump, check that the tube that aspirates the recording ACSF is not too close to the bubblestone, as this may lead to air bubbles flowing into the recording chamber, which can in turn introduce noise and artifacts to your recording.
- Monitor the flow rate and set it to 2-4 mL/min during the experiment. It is also good practice to measure the flow by collecting liquid from the outflow for a minute and comparing the volume you get with the flow you have set in the system.
- Measure the time it takes for the solution to actually reach the recording chamber - this will be useful to know if bath-applying drugs in your experiments.
- If using a harp, immerse it in the recording ACSF solution before placing a slice in the recording chamber. Otherwise, if you try to place it completely dry it may repel the recording ACSF (and sometimes even the slice you are trying to hold in place).
- When transferring a new brain slice into the bath make sure there are no air bubbles in the tissue, or it may float away instead of sinking to the bottom of the recording chamber. Once submerged, carefully position the slice with forceps or a brush by gently pushing from sides, without pinching/pressing the tissue and avoiding any unnecessary mechanical stress.
- Place the harp on the slice making sure that the closed U end is facing the incoming flow. This will help minimise any disturbances on your recording by the inflow. Furthermore, make sure the nylon strings are at the right distance and have the appropriate tension to hold the tissue without cutting through it.

9.3 [Replacing the coverslip in the recording chamber]

- Making a new recording chamber (i.e. replacing the coverslip at the bottom) requires an overnight step to let the sealant harden, so we recommend to always have a spare one ready to go in the unlikely (or unavoidable) event of breaking the coverslip at the bottom of the recording chamber by crashing the objective on it.
- If, or when, that happens, make sure to stop the pump as soon as possible to minimise any spillage of ACSF on the condenser or any other optic elements. If you manage to stop it in time and have a replacement chamber, you can simply replace the broken one with the spare one, reposition the ground/inflow/outflow/thermistor, transfer a new slice, and continue with your experiments. If the solution has reached the condenser, remove it to clean it right away (otherwise the ACSF will dry up and the salts will be harder to clean), dry it, place it back, and re-do the Kohler (see Section 9.1).
- To fix the broken chamber, start by removing any sealant and coverslip with a blade and thoroughly clean the chamber with 70% ethanol and ddH_2O.

Citation: Oriol Pavon Arocas, Tiago Branco Preparation of acute midbrain slices containing the superior colliculus and periaqueductal gray for patch-clamp recordings https://dx.doi.org/10.17504/protocols.io.8epv51pz5l1b/v6

This is an open access protocol distributed under the terms of the **Creative Commons Attribution License** (https://creativecommons.org/licenses/by/4.0/), which permits unrestricted use, distribution, and reproduction in any medium,

- Once it is clean and dry, use a cotton bud to apply some sealant on the edges (e.g. Dow Corning 732 multi-purpose sealant, Figure S15). Carefully mount a new coverslip and gently apply pressure to seal the gap between the chamber and the coverslip.
- With a new cotton bud, clean the excess sealant without smearing the coverslip (the light still needs to go through it to reach the brain slice), and leave the sealant to harden overnight.
- On the following morning, check that there is no leak by adding some water to the chamber. Use ddH20 and a kimwipe to clean the coverslip and store the newly fitted recording chamber as a spare.

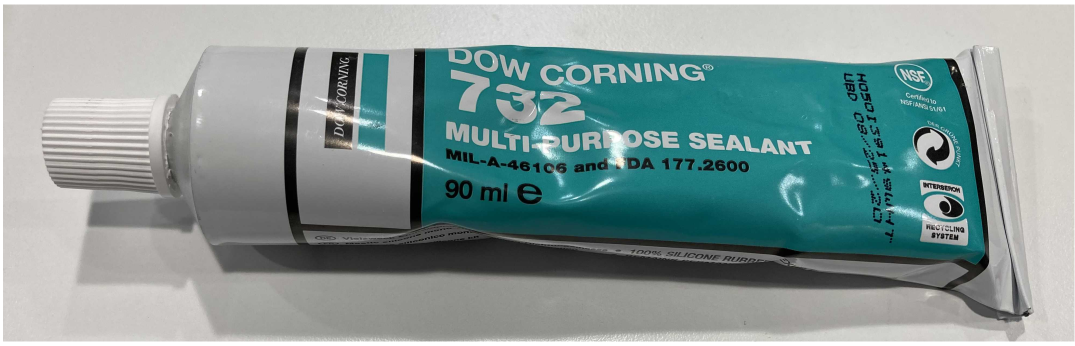

Figure S15. Dow Corning 732 multi-purpose sealant.

9.4 [Chloriding the recording and ground electrodes]

In our system, a silver wire (0.35 mm diameter, Cat. No. AG005145, GoodFellow) coated with silver chloride (AgCl) is present inside the patch pipette and in contact with the intracellular solution, and either an Ag-AgCl pellet electrode or a coiled AgCl coated silver wire is used as bath electrode (Figure S14). The extracellular and intracellular solutions contain chloride ions to allow a reversible exchange of ions: Ag + Cl- <-> AgCl + e-. To ensure that this ionic exchange occurs at all times, the silver wires need to be regularly chlorided. We found that chloriding the electrodes with solid silver chloride powder was the most stable and long-lasting method of the ones we tried.

- **Chloriding with solid silver chloride powder**: wearing gloves, clean the silver wire (you can carefully scrape off any dirt or residues with a blade). Scoop a bit of silver chloride powder (Cat. No. 204382-5G, Sigma Aldrich) with a glass pipette (the same type you use to make the pipette to pick up and transfer slices from one place to another, Figure S16) and hold over the flame of a bunsen burner until it melts (the powder will become liquid and turn into a dark brown colour). Once it is melted, slide the tip of the silver wire quickly in and out so it gets coated with the liquid silver chloride. Once it solidifies, you have a solid coating that is more stable and long-lasting than the other methods below.
- In order to avoid having to constantly re-chloride the ground (which will be

Citation: Oriol Pavon Arocas, Tiago Branco Preparation of acute midbrain slices containing the superior colliculus and periaqueductal gray for patch-clamp recordings https://dx.doi.org/10.17504/protocols.io.8epv51pz5l1b/v6

This is an open access protocol distributed under the terms of the **Creative Commons Attribution License** (https://creativecommons.org/licenses/by/4.0/), which permits unrestricted use, distribution, and reproduction in any medium,

necessary if using a coiled silver wire as a ground), a **Ag-AgCl pellet** and wire electrodes can be used (Cat. No. E206, Warner Instruments). These are more stable than the coiled silver wire and don't need to be rechlorided every now and then. Once the ground or baseline turns unstable, all you have to do is replace the pellet for a new one (as with the recording chamber, it is a good idea to have a spare ground pellet already soldered to a cable and pin connector compatible with the headstage ready to go).

- If you don't have ground pellets, you can make a ground with silver wire, but you will have to bleach/chloride it every now and then. Take a silver wire, fold it in half, and then folding it again. Roll it on its own and flame the tip until it melts and makes a tiny ball. You can then chloride/bleach it, solder it to a cable and pin connector compatible with the headstage, and use it as your ground electrode.

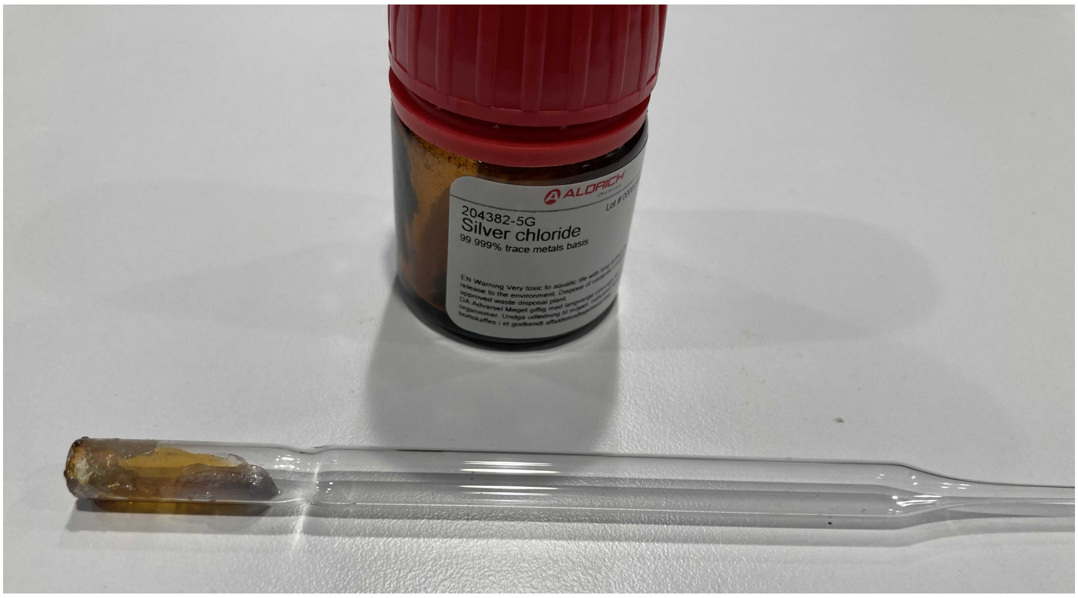

Figure S16. Glass pipette used to melt silver chloride powder over the flame of a bunsen burner.

Alternative ways of chloriding the electrodes are:

- **Chloriding with bleach**: pour some bleach on an eppendorf tube that has a hole in the cap, insert the electrode, and leave overnight. Once properly chlorided it should change to a darker colour (the Cl- has then correctly deposited on the surface of the silver wire).
- **Chloriding with 3 M KCL solution and a battery**: prepare a 3 M KCl solution in a bijou vial, place on the machine (Figure S17), connect the electrode you want to chloride to the crocodile clip above and a piece of cut silver wire to the other, place both electrodes in the 3 M KCl solution making sure they are making contact with their respective crocodile clips but not with each other. Pull the switch and let it run. It will switch off automatically once it is done after a few minutes. Again, if properly chlorided the electrode should have changed to a darker colour.

Citation: Oriol Pavon Arocas, Tiago Branco Preparation of acute midbrain slices containing the superior colliculus and periaqueductal gray for patch-clamp recordings https://dx.doi.org/10.17504/protocols.io.8epv51pz5l1b/v6

This is an open access protocol distributed under the terms of the **Creative Commons Attribution License** (https://creativecommons.org/licenses/by/4.0/), which permits unrestricted use, distribution, and reproduction in any medium,

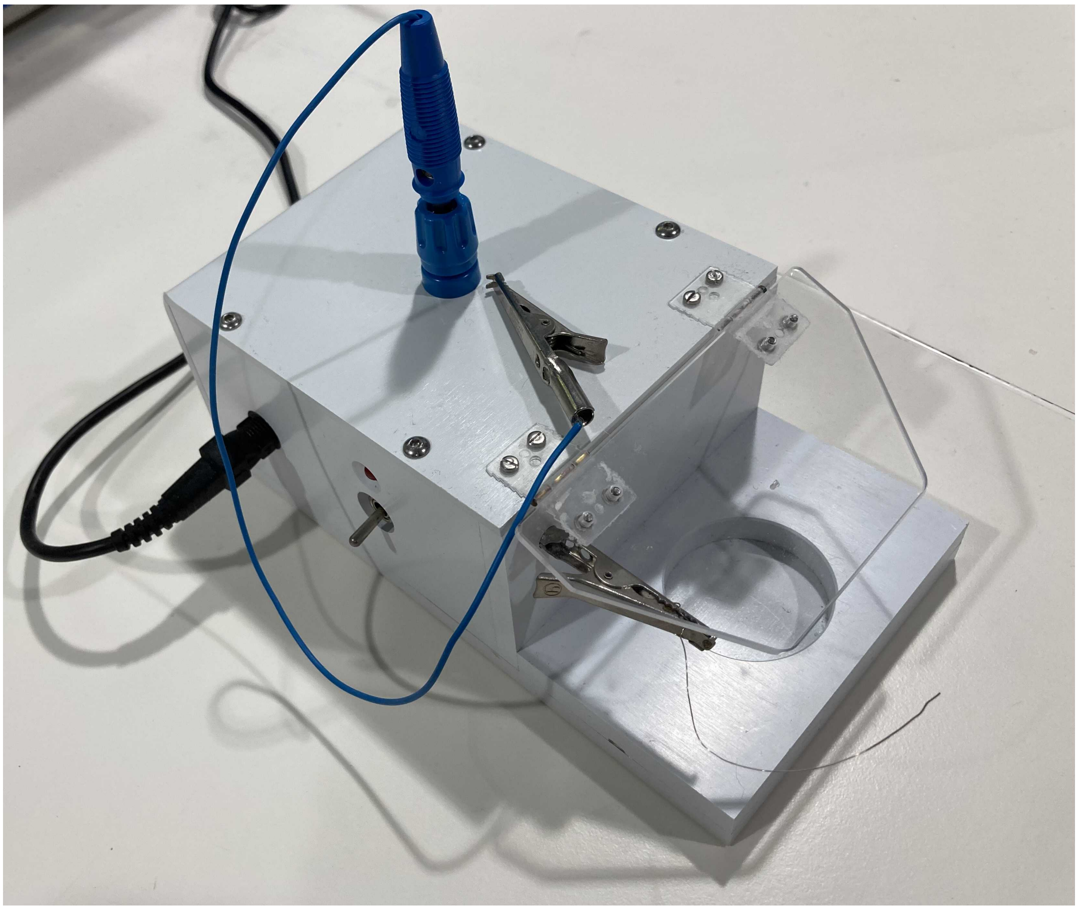

Figure S17. Example of a machine that can be used to chloride electrodes.

9.5 [Pipette holder]

- It is good practice to periodically take apart the holder to clean all the parts and replace the O-rings if necessary.
- Use gloves when taking it apart and assembling it back, to avoid depositing any grease from your skin onto the materials.
- Clean all the parts with ddH2O and let dry overnight. Once dry, use an air duster to clean and remove any dust or water left.
- Re-assemble the holder [if using a HEKA pipette holder, see link & link].

9.6 [Setting up and adjusting a micromanipulator]

- Angle the micromanipulator to around 15-20 degrees and center it across all axis (i.e. if the axis can go to 25000, set it to 12500). To check it has been properly set up and adjusted, mount a patch pipette on the holder and position the manipulator over the bath chamber. The tip of the pipette should now stand in the center of the bath chamber. This should give you maximal mobility across all axes.

Citation: Oriol Pavon Arocas, Tiago Branco Preparation of acute midbrain slices containing the superior colliculus and periaqueductal gray for patch-clamp recordings https://dx.doi.org/10.17504/protocols.io.8epv51pz5l1b/v6

This is an open access protocol distributed under the terms of the **Creative Commons Attribution License** (https://creativecommons.org/licenses/by/4.0/), which permits unrestricted use, distribution, and reproduction in any medium,

- The tubing that connects to the pipette holder for applying pressure and suction should be as short as possible, and ideally made of hard material. A more elastic tube will give way and expand when pressure is applied, potentially leading to small amounts of pressure loss as well as increasing the time it takes for the suction pulses to reach the cell membrane when breaking in.

9.7 [Harp]

- The harp is usually a U-shaped piece of platinum wire with a few nylon strings evenly spaced (reasonably frequent and not too close or too far apart) that hold the brain slice in place so that you can achieve recordings without the tissue being moved by the constant flow of ACSF or the pipette.
- To make a harp, you can use 1 mm diameter platinum wire (0.1 m length, Cat. No. PT005155, GoodFellow) which has been flattened, cut, and shaped to the desired dimensions.
- The nylon strings can then be positioned on one of the flattened sides with the preferred separation and tension, and fixed to the platinum wire using superglue (e.g. Universal, Loctite).

9.8 [Backfilling glass electrodes]

- You can use a 2-20 µL pipette and microloader tips to fill microcapillaries (i.e. patch pipettes) with internal solution.
- Use enough solution to cover the tip of the silver wire that acts as recording electrode in the headstage, but don't overfill as this will increase the capacitance of your pipette.
- Instead of microloader tips, it is also possible to use a lighter to heat a sterile 200 µL tip until it begins to melt, and then manually pull your own thin tip to backfill the glass electrodes.

9.9 [Liquid Junction potential]

Whenever you have different solutions in the recording pipette and the bath, there will be a charge separation across the junction between the two solutions, owing to the different ionic composition of each solution. This charge separation will result in what is commonly known as liquid junction potential. You can either calculate the expected liquid junction potential for your two solutions or measure it empirically.

Useful references to learn more about the liquid junction potential and how to measure it:

- Neher E. Correction for liquid junction potentials in patch clamp experiments. Methods in Enzymology. 1992;207(8), pp. 123–31. DOI: https://doi.org/10.1016/0076-6879(92)07008-C
- "What is a junction potential?", blog by Nat Blair [link].

Citation: Oriol Pavon Arocas, Tiago Branco Preparation of acute midbrain slices containing the superior colliculus and periaqueductal gray for patch-clamp recordings https://dx.doi.org/10.17504/protocols.io.8epv51pz5l1b/v6

This is an open access protocol distributed under the terms of the **Creative Commons Attribution License** (https://creativecommons.org/licenses/by/4.0/), which permits unrestricted use, distribution, and reproduction in any medium,

- "How do I measure the Liquid Junction Potential?", blog by Nat Blair [link].
- Liquid Junction Potential Calculator, code from Scott W Harden [GitHub] adapted from Marino, et al. A new open source software for the calculation of the liquid junction potential between two solutions according to the stationary Nernst-Planck equation. arXiv. 2014. DOI: https://arxiv.org/abs/1403.3640

9.10 **[Bubblestones]**

- Should you notice that mould starts to grow on the tip of a bubblestone, you can try to clean it by leaving it immersed in H2O2 overnight. On the next morning, thoroughly clean with ddH2O and leave all day in ddH2O. Finally, rinse a bit more to make sure any H2O2 residues are gone before using them again.
- Alternatively, replace for a new one.

Citation: Oriol Pavon Arocas, Tiago Branco Preparation of acute midbrain slices containing the superior colliculus and periaqueductal gray for patch-clamp recordings https://dx.doi.org/10.17504/protocols.io.8epv51pz5l1b/v6

This is an open access protocol distributed under the terms of the **Creative Commons Attribution License** (https://creativecommons.org/licenses/by/4.0/), which permits unrestricted use, distribution, and reproduction in any medium,
